# HIF activation enhances FcγRIIb expression on mononuclear phagocytes impeding tumor targeting antibody immunotherapy

**DOI:** 10.1186/s13046-022-02294-5

**Published:** 2022-04-07

**Authors:** Khiyam Hussain, Rena Liu, Rosanna C. G. Smith, Kri T. J. Müller, Mohammadmersad Ghorbani, Sofia Macari, Kirstie L. S. Cleary, Robert J. Oldham, Russell B. Foxall, Sonya James, Steven G. Booth, Tom Murray, Lekh N. Dahal, Chantal E. Hargreaves, Robert S. Kemp, Jemma Longley, James Douglas, Hannah Markham, Serena J. Chee, Richard J. Stopforth, Ali Roghanian, Matthew J. Carter, Christian H. Ottensmeier, Bjorn Frendéus, Ramsey I. Cutress, Ruth R. French, Martin J. Glennie, Jonathan C. Strefford, Stephen M. Thirdborough, Stephen A. Beers, Mark S. Cragg

**Affiliations:** 1 Antibody and Vaccine Group, Centre for Cancer Immunology, School of Cancer Sciences, Faculty of Medicine, University of Southampton, Tremona Road, Southampton, SO16 6YD UK; 2 Cancer Genomics Group, Southampton Experimental Cancer Medicine Centre, School of Cancer Sciences, Faculty of Medicine, University of Southampton, Southampton, SO16 6YD UK; 3 Nuffield Department of Medicine, John Radcliffe Hospital, University of Oxford, Oxford, OX3 9DU UK; 4 University Hospital Southampton, Southampton General Hospital, Tremona Road, Southampton, SO16 6YD Hampshire UK; 5 CRUK Southampton Centre, School of Cancer Sciences, Faculty of Medicine, University of Southampton, Tremona Road, Southampton, SO16 6YD UK; 6 Preclinical Research, BioInvent International AB, Sölvegatan 41, 22370 Lund, Sweden

**Keywords:** Hypoxia, Hypoxia inducible factors, FcγRIIb, Fc gamma receptors, Tumor-associated macrophages, Monocytes, Monoclonal antibody, Tumor microenvironment, Resistance, Cancer

## Abstract

**Background:**

Hypoxia is a hallmark of the tumor microenvironment (TME) and in addition to altering metabolism in cancer cells, it transforms tumor-associated stromal cells. Within the tumor stromal cell compartment, tumor-associated macrophages (TAMs) provide potent pro-tumoral support. However, TAMs can also be harnessed to destroy tumor cells by monoclonal antibody (mAb) immunotherapy, through antibody dependent cellular phagocytosis (ADCP). This is mediated via antibody-binding activating Fc gamma receptors (FcγR) and impaired by the single inhibitory FcγR, FcγRIIb.

**Methods:**

We applied a multi-OMIC approach coupled with in vitro functional assays and murine tumor models to assess the effects of hypoxia inducible factor (HIF) activation on mAb mediated depletion of human and murine cancer cells. For mechanistic assessments, siRNA-mediated gene silencing, Western blotting and chromatin immune precipitation were utilized to assess the impact of identified regulators on *FCGR2B* gene transcription.

**Results:**

We report that TAMs are FcγRIIb^bright^ relative to healthy tissue counterparts and under hypoxic conditions*,* mononuclear phagocytes markedly upregulate FcγRIIb. This enhanced FcγRIIb expression is transcriptionally driven through HIFs and Activator protein 1 (AP-1). Importantly, this phenotype reduces the ability of macrophages to eliminate anti-CD20 monoclonal antibody (mAb) opsonized human chronic lymphocytic leukemia cells in vitro and EL4 lymphoma cells in vivo in human FcγRIIb^+*/*+^ transgenic mice. Furthermore, post-HIF activation, mAb mediated blockade of FcγRIIb can partially restore phagocytic function in human monocytes.

**Conclusion:**

Our findings provide a detailed molecular and cellular basis for hypoxia driven resistance to antitumor mAb immunotherapy, unveiling a hitherto unexplored aspect of the TME. These findings provide a mechanistic rationale for the modulation of FcγRIIb expression or its blockade as a promising strategy to enhance approved and novel mAb immunotherapies.

**Supplementary Information:**

The online version contains supplementary material available at 10.1186/s13046-022-02294-5.

## Background

Hypoxia is a state that arises when cellular demand for molecular oxygen (O_2_) exceeds supply [1]. Several studies have reported that hypoxia is a distinctive aspect of a wide range of solid tumors [2–9] and over half of tumor regions exhibit lower O_2_ levels relative to their healthy tissue counterparts [10]. In the atmosphere, *pO*_*2*_ is 160 mmHg (21.1%), falling to 100 mmHg (13.2%) in arterial blood [11]. In comparison, in pancreatic ductal adenocarcinoma, median *pO*_*2*_ is 0–5.3 mmHg (0–0.7%) compared to 24.3–92.7 mmHg (3.2–12.3%) in donor matched healthy pancreas [5]. Cells respond to hypoxia by stabilizing the hypoxia-inducible factor (HIF) family of transcription factors. In the tumor microenvironment (TME) the genes induced by HIF-1α and HIF-2α enhance tumor growth and survival, by increasing angiogenesis, cell survival, cell proliferation, metastasis, pH regulation, glycolysis and maintenance of cancer stem cells [12].

Among the diverse cell populations present in the TME, macrophages are often the most abundant and are referred to as tumor-associated macrophages (TAMs) [13]. Macrophages exist in multiple states of activation with so-called M1 and M2 describing their extremes; M1 macrophages (generated through LPS/IFN-γ stimulation) are pro-inflammatory and are thought to possess anti-tumor functions; M2 macrophages (produced following Interleukin (IL)-4/IL-13 treatment) are considered anti-inflammatory and pro-tumor [14, 15]. Although TAMs are thought to acquire a primarily proangiogenic tumor promoting (M2-like) phenotype in the TME [16].

Clinically important tumor targeting monoclonal antibodies (mAb) such as Rituximab, Herceptin and Cetuximab, function, at least in part, by inducing mononuclear phagocytes to deplete tumor cells [18–23]. Furthermore, mAbs such as Ipilimumab, targeting immune checkpoint molecules, previously thought to function solely via receptor blockade and expansion of effector T (Teff) cells [24], have also recently been reported to work optimally through myeloid-cell mediated depletion of tumor infiltrating immunosuppressive regulatory T (Treg) cells [25–27].

A key mechanism by which direct targeting anti-cancer mAbs deplete cellular targets in the TME is via antibody dependent cellular phagocytosis (ADCP) which is primarily accomplished by macrophages [28]. As such, mAb-bound target cells interact with the activating Fc gamma receptors (FcγRs); FcγRI, FcγRIIa and FcγRIIIa for optimal ADCP (FcγRI, FcγRIII and FcγRIV in the mouse), whereas engagement with the sole inhibitory FcγR, FcγRIIb (FcγRII in mice) attenuates phagocytic function [29]. Expression levels and cellular distribution of FcγR on effector cells are therefore of crucial importance in antibody therapy outcome.

Although an important feature of many tumors, the impact of physiological hypoxia on anti-cancer mAb immunotherapy has not been investigated in detail to date. In the current study we applied a multi-OMIC approach to profile the effects of hypoxia on FcγR expression in mononuclear phagocytes and its subsequent impact on antitumor mAb effector functions. We demonstrate that exposure to physiological or pharmaceutical hypoxia, induces transcriptionally driven and rapid upregulation of FcγRIIb expression on mononuclear phagocytes. Hypoxia-mediated enhancement of FcγRIIb expression impairs ADCP and reduces in vivo therapeutic mAb efficacy in murine tumor models. We provide a detailed molecular and cellular basis for tumor hypoxia driven resistance to mAb immunotherapy, unveiling a hitherto unexplored aspect of the TME that requires evaluation for current and novel mAb immunotherapies to improve clinical efficacy.

## Methods

### Human subjects

Anonymized leukocyte cones were sourced from healthy adult donors attending blood donation clinics at the National Blood Service (Southampton, UK). Peripheral blood mononuclear cells (PBMCs), primary monocytes and T cells, were then isolated from these leukocyte cones for molecular characterization and functional assays to determine the effects of hypoxia on FcγR expression and IgG effector functions. The use of leukocyte cones for this work was approved by the University of Southampton Faculty of Medicine Ethics Committee and the East of Scotland Research Ethics Service, Tayside, UK, Research ethical committee (REC) reference number: 16/ES/0048. To evaluate FcγR expression on monocytes and macrophages (mo/mθ) in cancer patients, donor matched whole peripheral blood (5–10 mL) and pleural fluid samples (50–400 mL) were sourced from 6 anonymized mesothelioma patients (REC reference number: 13/SW/0128). Donor matched Renal cell carcinoma (RCC) and non-cancerous healthy kidney tissue samples were obtained from resected kidneys from 5 RCC patients (REC reference number: 17/WA/0241). Lymphocele samples (30–100 mL) were sourced from 3 anonymized breast cancer patents (REC reference number: 10/H0504/73, for breast cancer patient samples). Peripheral blood samples were taken from Chronic lymphocytic leukemia (CLL) patients, PBMCs were isolated and placed in 90% Fetal calf serum (FCS)/10% Dimethyl sulfoxide (DMSO; Sigma-Aldrich) and stored in liquid nitrogen until further use as target cells in monocyte-derived macrophage (MDM) based phagocytosis assays (REC reference number: 10/H0504/187, for CLL patient samples). These aforementioned clinical samples were released from the Human Tissue Authority Licensed University of Southampton, Cancer Sciences Tissue Bank, as approved by the Southampton and South West Hampshire Research Ethics Committee (REC reference: 280/99). All informed consent for the use of human material was provided in accordance with the Declaration of Helsinki.

### Mice

Mice were used in these studies as the least sentient species with an immune system comparable to humans. The availability of a transgenic (Tg) mouse strain expressing human (h) FcγRIIb also facilitates more detailed understanding of the effects of hypoxia on mAb mediated cell target depletion in a living organism to inform clinical translation. Wild type (WT) C57BL/6 and hFcγRIIB^+/−^ x mouse (m) FcγRII^−/−^ x hCD20^+/−^ C57BL/6 J mice were described previously [30] and were maintained and bred in house. Splenocytes from hCD20^+/−^ x mFcγRII^−/−^ were used as target cells in the adoptive transfer in vivo experiment in Fig. 7i-j (NB: mFcγRII^−/−^ cells were selected to remove any potential influence from mFcγRII changes on the target cells). Genotypes were confirmed by PCR and/or flow cytometry. All mice were bred in a closed research facility under specific pathogen-free conditions in individually ventilated cages (IVCs). Following approval by local ethical committees, reporting to the Home Office Animal Welfare Ethical Review Board (AWERB) at the University of Southampton, in vivo experiments were conducted under UK Home Office Project licenses P81E129B7 and P4D9C89EA. Experiments used both male and female mice, and mice were age and sex matched within experiments. For the majority of experiments mice were aged between 8–15 weeks. Littermates of the same sex were randomly assigned to experimental groups at the start of the experiment. Mice were maintained on a 12-h light/dark cycle, food and water was made available at all times, environmental enrichment was provided, and temperature was maintained between 20–24 °C. Mice were visually checked daily if adverse effects were anticipated or if mice were nearing a humane end-point.

### Isolation of murine immune cells

To prepare myeloid cells from murine spleens for flow cytometric analysis, harvested tissue was cut into small pieces, placed in 5 mL complete RPMI (RPMI-1640 supplemented with, 2 mM L-glutamine, 1 mM pyruvate, 100 U/mL penicillin, 100 mg/mL streptomycin and 10% heat inactivated HyClone FCS (GIBCO)) and mechanically dissociated via feeding the tissue through a 70 μm BD Falcon cell filter (BD Biosciences) to achieve a single cell suspension. 1 mL of ammonium chloride buffer (154.4 mM ammonium chloride and 10 mM potassium bicarbonate (Sigma-Aldrich)) was added to lyse red blood cells (RBCs) in each spleen sample and samples were washed once in complete RPMI. To isolate immune cells from the peritoneum, mice were sacrificed, and 5 mL of ice-cold phosphate buffered saline (PBS) was injected into the peritoneum using a 10 mL syringe and a 25-G needle. The peritoneum was then gently massaged, and the PBS drawn back into the syringe to achieve a single cell suspension. To prepare bone marrow immune cells for flow cytometric analysis, mice were sacrificed and hind femora and tibiae isolated. Muscle and soft tissue were removed from the bones and each bone trimmed at both ends. Bone marrow was then flushed with complete RPMI until bones were white. The liberated cells were passed through a 70 μm BD Falcon cell filter (BD Biosciences) to achieve a single cell suspension. All murine livers and tumors were cut into small 2 mm × 2 mm pieces, incubated in 1.5 mL serum free RPMI 1640 per liver or 500 mm^3^ tumor, with 200 U/mL DNase I (Sigma-Aldrich) and 15 U/mL Liberase TL (Roche Diagnostics) for 30 min, at 37 °C in a shaking incubator. 25 mL complete RPMI was added per sample and the digested samples were mechanically dissociated and, together with the cell suspension, passed through a 70 μm BD Falcon cell filter and centrifuged (300 × *g* for 5 min). Additionally, immune cells in liver samples were separated from non-immune cells via Percoll (Sigma-Aldrich) density centrifugation [31]. The supernatants from each tumor and liver sample were removed and 5 mL of ammonium chloride buffer added to lyse RBCs. Post-isolation, all murine tumor, liver, splenocyte, peritoneal lavage and bone marrow single cell suspensions were centrifuged (300 × g for 5 min) and resuspended in complete RPMI at 1 × 10^7^ cells/mL, prior to staining with fluorophore conjugated antibodies.

### Isolation of human immune cells

PBMCs were isolated from leukocyte cones or from whole peripheral blood from mesothelioma patients, within 2 h of collection, by density gradient centrifugation at 800 × *g* for 20 min (Lymphoprep, Axis-Shield). Primary human monocytes were isolated using the Pan Monocyte Isolation Kit, human (Miltenyi Biotech) from 1 × 10^8^ PBMCs per isolation, according to the manufacturer’s protocol.

Pleural fluid and lymphocele clinical samples were centrifuged at 300 × *g* for 10 min and the supernatants were removed. RBCs were lysed with Erythrolyse Red Blood cell (RBC) lysis buffer (AbD SeroTec) and samples washed once in PBS/1% Bovine Serum Albumin + 10% FCS (Sigma-Aldrich) and stained immediately with fluorophore conjugated mAbs for flow cytometric analysis.

RCC or healthy (normal) kidney tissue (0.9–1.3 g of tissue per sample) was cut into small 2 mm × 2 mm pieces, incubated in 1.5 mL RPMI 1640 per 0.3 g of tissue, with 200 U/mL DNase I (Sigma-Aldrich) and 15 U/mL Liberase TM (Roche Diagnostics) for 45 min, at 37 °C in a shaking incubator. 25 mL of complete RPMI was added per 0.3 g of tissue and the digested samples mechanically dissociated and, together with the cell suspension, passed through a 70 μm BD Falcon cell filter (BD Biosciences) and centrifuged (300 × *g* for 5 min). The supernatant was removed and 5 mL of ammonium chloride buffer added to lyse RBCs. Each sample was then centrifuged (300 × *g* for 5 min), the supernatant discarded, the cell pellet resuspended in complete RPMI with 10% Human AB serum (Invitrogen) at 1 × 10^7^ cells/mL and incubated at room temperature for 15 min. These cell suspensions were then centrifuged (300 × *g* for 5 min), the supernatants discarded and resuspended in complete RPMI and stained immediately with fluorophore conjugated mAbs for flow cytometric analysis.

### Cell lines and transfections

Chinese hamster ovary (CHO) K1 (ATCC CCL-61) cells were cultured in complete RPMI 1640 with 0.05 mM β-mercaptoethanol (2ME; Sigma-Aldrich) and incubated at 37 °C, 5% CO_2_. CHO-K1 cells were then transfected with FcγRIIb1 or FcγRIIb2 isoforms in plasmid pcDNA3 [32], selected by using 1 mg/mL geneticin (Life Technologies), and screened by flow cytometry using the pan-FcγRII mAb AT10 F(ab’)_2_-FITC (in-house). Positive colonies were expanded and then sorted using a FACSAria II flow cytometer (BD Biosciences). THP-1 (human leukemia monocytic cell line; ATCC TIB-202) cells were obtained from LGC Standards (Middlesex, UK). THP-1 cells were cultured in complete RPMI 1640 medium with 0.05 mM 2-ME, maintained at 0.25–0.5∙10^6^ cells/ml and incubated at 37 °C, 5% CO_2_. THP-1 cells were passaged before reaching 1 × 10^6^ cells/ml. EL4 cells (ATCC® TIB-39™), a murine thymoma cell line, were cultured in complete RPMI 1640 supplemented with 0.05 mM 2-ME. Cells were maintained between 1 × 10^5^ and 1 × 10^6^ cells/mL and incubated at 37 °C, 5% CO_2_. These EL4 cells were then transfected with human CD20 in plasmid pcDNA3 [33], selected with 10 μg/mL puromycin (GIBCO). Human CD20 expression was screened by flow cytometry, using Rituximab (Roche) conjugated in-house using an Alexa Fluor™ 488 Protein Labelling Kit (ThermoFisher Scientific). Positive colonies were expanded and then sorted using a FACSAria II flow cytometer (BD Biosciences). MC38 (murine colon adenocarcinoma cell line, kindly gifted by Dr Sjef Verbeek), MCA205 (fibrosarcoma cell line, Sigma-Aldrich) and E.G7-OVA (T cell lymphoma cell line expressing model antigen hen egg ovalbumin, ATCC®, CRL-2113™) were maintained in complete RPMI supplemented with 0.05 mM 2-ME. E0771 (breast carcinoma cell line, ATCC®, CRL-3461™) cells were maintained in complete RPMI alone.

### Antibodies

In the human monocyte phagocytosis assays, Rhesus D antigen positive RBCs were used as target cells via opsonization with wild type human IgG1 anti-Rhesus D mAb, kind-gift from Dr Gestur Vidarsson (Sanquin Blood Supply Foundation, Amsterdam, Netherlands). In Fig. 7c, to block IgG Fc-FcγRII interactions, E08 (anti-human-FcγRIIa) or 6G11 (anti-FcγRIIb), described previously [30, 34], provided by BioInvent International AB (Lund, Sweden), were used as F(ab’)_2_ fragments to pre-treat primary human monocytes, before assessment of RBC ADCP capacity. F(ab’)_2_ fragments were produced by trypsin digestion as before [35]. mAb was purified using Protein A-Sepharose, and aggregates were removed by gel filtration. Preparations were endotoxin low (< 1 ng/mg protein, Endosafe-PTS, Charles River Laboratories). For the human MDM ADCP assays, and when targeting human CD20^+^ target cells in vivo, clinical grade Rituximab (Roche) and Obinutuzumab (Roche) were used (kindly gifted by Prof Christian Klein, Roche). Cetuximab (Merck KGaA) and Herceptin (Roche, kindly gifted by Thomas Valerius) were used as isotype controls for the aforementioned anti-CD20 mAbs. For systemic B cell depletion in wild type C57BL/6 J mice in Additional file 7: Fig. S7h, anti-murine CD20 antibody, clone 18B12 (produced in-house) was used as a mouse IgG2a to systemically deplete B cells as previously described [36, 37].

### Generation of monocyte-derived macrophages (MDMs)

PBMCs were isolated from leukocyte cones from healthy donors and were seeded 2 × 10^7^ cells per well in 6-well plates (Corning Centristar), in complete RPMI (supplemented with 1% human AB serum, Invitrogen) at 37 °C and 5% CO_2_ for 2 h. Non-adherent cells were removed with PBS and cells cultured overnight in complete RPMI. Adherent monocytes were then differentiated with M-CSF (100 ng/ml) on days 2, 4 and 6 with or without Dimethyloxalylglycine (DMOG) or Roxadustat (both at 20 µM). On Day 7 post-culture, MDMs were left untreated or stimulated with IFN-γ (50 ng/mL) and LPS (2 ng/mL), or IL-4 (20 ng/mL) and IL-13 (10 ng/mL), to generate M0, M1 and M2-like macrophages, respectively. Changes in cell morphology were assessed by phase contrast microscopy (Axiovert 135, Zeiss). Phenotypic and functional characterization of MDMs was performed 9 days post-culture: MDMs were removed from the plates with a cell scraper, 2 × 10^5^ cells were transferred to each FACS tube and analyzed using BD FACSCantoII or FACSCalibur (BD Biosciences) flow cytometers and FlowJo Version 10 software (FlowJo LLC).

### Cell culture

**Fig. 1:**
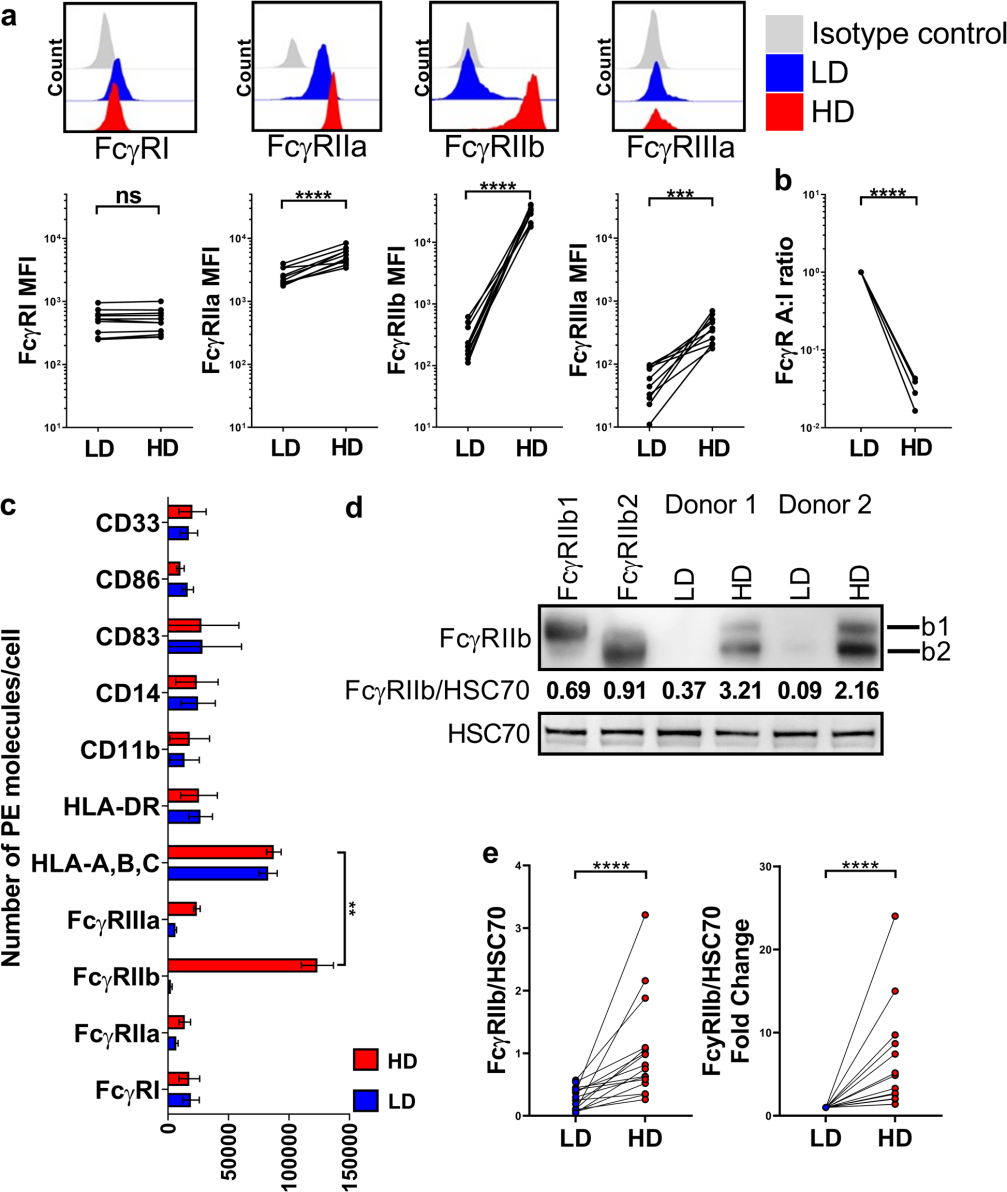
FcγR expression profiling of human PBMCs cultured at low or high density for 48 h. **a**, Expression of FcγR on primary human monocytes (FSC^hi^CD14^+^ cells) in low density (LD) or high density (HD) PBMC cultures determined using flow cytometry. Representative histograms above and quantified for 11 independent healthy donors below. **b**, Comparison of FcγR activating:inhibitory (A:I) ratio between LD and HD monocytes, (*n* = 11 per group). **c**, Quantification of FcγR and myeloid cell surface markers on monocytes in LD and HD PBMC cultures determined using flow cytometry and PE fluorescence quantitation beads, group means ± SD are shown, (*n* = 5 per group). **d**, Assessment of FcγRIIb expression by Western blot in LD and HD monocytes using CHO cells transfected with FcγRIIb1, and FcγRIIb2 isoforms as controls. **e**, Combined Western blot data of FcγRIIb expression normalized to HSC70 loading control (left) and fold change of FcγRIIb expression relative to HSC70 in LD and HD monocytes (right), (*n* = 16 per group). Each data point represents a unique healthy adult donor. Statistical significance between groups was assessed using a paired two-tailed Wilcoxon test (***p < 0.001, ****p < 0.0001 and ns = non-significant). Also see Additional file 1: Fig. S1

In Fig. 1, PBMCs from heathy human subjects were cultured at low density (LD) defined as 1 × 10^6^ cells/mL or at high density (HD) defined as 1 × 10^7^ cells/mL, in serum free complete CTL-Test Medium ((Europe GmbH, Bonn, Germany), supplemented with glutamine (2 mM), pyruvate (1 mM), penicillin (100 IU/mL), and streptomycin (100 IU/mL)), at 37 °C in 5% CO_2_. 1.5 mL of cells at the aforementioned concentrations were cultured in flat bottomed 24-well plates (Corning Centristar) for 48 h before immunophenotyping using flow cytometry and Western blotting.

To determine whether physiological hypoxia leads to FcγRIIb upregulation on human monocytes, LD PBMCs or purified monocytes were incubated in a hypoxic chamber (Billups-Rothenberg, Inc). Cells were cultured in complete CTL medium in a 24-well plate and placed in the hypoxic chamber. To create hypoxic conditions, the chamber was attached to a gas cylinder containing 1% O_2_, 5% CO_2_ and 94% N_2_ with tubing using a flow meter incorporated in a regulator. The chamber was gassed at a flow rate of 20 L/minute for 7–10 min and then sealed whilst being filled with 1% O_2_. This step was repeated an hour later. Cells in the sealed hypoxic chamber were then kept inside a conventional incubator at 37 °C for 48 h before FcγR expression levels were assessed using flow cytometry. PBMCs or purified monocytes were also cultured at 3% O_2_ levels in complete CTL medium, in a Thermo Scientific tri-gas incubator (Thermo Fisher Scientific), at 37 °C and 5% CO_2,_ followed by FcγR expression assessment using flow cytometry.

To stabilize HIF-1α and HIF-2α in primary mononuclear phagocytes and THP-1 cells, HIF prolyl hydroxylase (HIF-PH) inhibitors were utilized as hypoxia mimetics. DMOG was purchased from EMD Millipore, directly dissolved in sterile PBS to a stock concentration of 20 mg/mL, then filter sterilized and stored aliquoted at -80 °C. Roxadustat (FG-4592) was purchased from Stratech Scientific Ltd, dissolved in DMSO to create 50 mg/mL stock solution aliquots; these were further diluted in sterile PBS to 1 mg/mL, and stored aliquoted at –80 °C. To determine whether the stabilization of hypoxia inducible factors leads to FcγRIIb upregulation on monocytes, human PBMCs, purified monocytes or MDMs were incubated with 20 µM DMOG or 20 µM Roxadustat in complete CTL medium, at 37 °C and 5% CO_2_ for 24 or 48 h, before flow cytometric analysis. THP-1 cells were treated with 0–200 µM DMOG.

To assess the role of HIFs and Activator protein 1 (AP-1) in the upregulation of FcγRIIb in human monocytes and MDMs the following reagents were utilized to impair function or reduce expression of HIFs and or AP-1 transcription factor complex protein; c-Jun. In Additional file 5: Fig. S5a-b HD PBMCs were treated with 10 µM Digoxin (Sigma-Aldrich). In Fig. 5i LD PBMCs were treated with VHL inhibitor; VH298 [38], at 25 µM (Sigma-Aldrich). In Fig. 5j LD PBMCs were simultaneously treated with 20 µM DMOG and 20 µM of the HIF-α inhibitor; FM19G11 [39], (Sigma-Aldrich). To impair JNK/c-Jun interactions in Fig. 5k, LD PBMCs were treated with 20 µM DMOG and were simultaneously treated with 1 mM c-Jun peptide (R&D Systems [40]). All inhibitor treated cells were cultured in complete CTL medium for 24 h, followed by FcγR expression assessment using flow cytometry. M1 MDMs were treated with c-Jun peptide with or without simultaneous treatment with 20 µM DMOG for 48 h. These M1 MDMs were cultured in complete RPMI 1640 with M1 skewing agents (IFN-γ and LPS) and incubated at 37 °C and 5% CO_2_, followed by FcγR expression assessment using flow cytometry.

### siRNA

For siRNA manipulation of monocytes, immediately after isolation from PBMC samples, monocytes were washed in PBS, centrifuged at 300 × *g* for 5 min, supernatant discarded and the cell pellet resuspended at 3 × 10^7^ cells/mL in buffer T (ThermoFisher). A NeonTM tube was filled with 3 mL of buffer E2 and inserted into the NeonTM Pipette Station. *HIF1Α*, *HIF2A*, *JUN* or Silencer*™* negative control siRNA (ThermoFisher) were added to the isolated monocyte/buffer T mixture so that the final working concentration of siRNA was 100 nM. The siRNA/monocyte/buffer T mixture was taken up into a 100 µL NeonTM Pipette tip (ThermoFisher) and electroporated with the Neon transfection system (ThermoFisher) using the settings: 1920 V, 25 ms, 1 pulse. The electroporated cells were either shared across 2 wells of a 24-well plate with 1 ml of antibiotic free media (CTL medium + 10% FCS) for LD culture; or placed in a single well of a 96-well plate with 100 µl antibiotic-free media for HD culture. Electroporated cells were incubated in antibiotic-free CTL medium for 48 h before FcγR expression was analyzed by flow cytometry.

### Flow cytometry of human cells

Human immune cells were first incubated in complete RPMI 1640 supplemented with 10% Human AB serum (Invitrogen) and incubated at room temperature for 15 min and then centrifuged at 300 × g for 5 min. Cells were then resuspended at 10 × 10^6^ cells/mL in flow cytometry wash buffer (PBS with 1% w/v BSA (Europa), 0.1% w/v sodium azide (Sigma-Aldrich)). 1 × 10^6^ PBMCs, purified monocytes, MDMs, THP-1 cells, immune cells isolated from pleural fluid, lymphocele, RCC or healthy (normal) kidney tissue in 100 µL were stained with fluorophore-conjugated mAbs per FACS tube, for 30 min at 4 °C. Samples were stained with anti-CD3 PerCP (clone: SK7), anti-CD56 PE (clone: HCD56), anti-CD19 APC-Cy7 (clone: HIB19), anti-CD14 Pacific Blue (clone: M5E2), anti-CD163 PE-Cy7 (clone: RM3/1), anti-HLA-DR APC-Cy7 (clone: L243), anti-CD40 APC-Cy7 (clone: 5C3), anti-human CD11b PE (clone: ICRF44), anti-human CD274 (B7-H1, PD-L1) PE (clone: MIH3) or IgG1κ-FITC (clone: MOPC-21) isotype control (all from BioLegend). FcγR staining was carried out using anti-FcγRI FITC (clone: 10.1, F(ab')_2_), anti-FcγRII PE (clones: AT10 and KB61), anti-FcγRIIa FITC (clone: E08, F(ab')_2_), anti-FcγRIIb FITC (clone: 6G11, F(ab')_2_), anti-FcγRIIb FITC (clone: 2B6, F(ab')_2_), anti-FcγRIIIa FITC (clone: 3G8, F(ab')_2_), and isotype control human IgG1 FITC (clone: FITC8 F(ab')_2_) were all generated from published sequences in-house or provided by BioInvent International AB. The FcγR activating:inhibitory (A:I) ratio was calculated by summing up the Geometric mean (Geomean) fluorescent intensities of the activating FcγR (FcγRI, FcγRIIa and FcγRIIIa) staining and dividing by the Geomean fluorescent intensity of FcγRIIb staining. Alternatively, anti-FcγR mAbs were used in an APC format. In order to determine expression levels of the hypoxia marker; Carbonic Anhydrase IX, anti-CAIX (clone: M75), mouse IgG1, Fc Silent™, Kappa APC (Absolute antibody) was used. Cells were then washed with flow cytometry wash buffer and if pre-cultured with translation, transcription, HIF-PHD, HIF-α or c-Jun inhibitors, cells were also stained with propidium iodide (PI, Sigma-Aldrich) before analysis of the cells by flow cytometry, in order to determine cell viability. HIF-1α and GLUT1 were measured using the Hif1α + GLUT1 Hypoxic Response Human Flow Cytometry Kit (Abcam). 1 × 10^6^ cells LD or HD monocytes were harvested and fixed in 4% paraformaldehyde (Sigma-Aldrich) for 15 min and pelleted. The pellet was resuspended in 90% ice cold methanol and incubated at -20 °C for at least 30 min. Within 1 week of fixing, cells were warmed to room temperature and the methanol removed before rehydrating and washing cells in blocking buffer. After incubating cells for 30 min in blocking buffer the primary mouse HIF-1α and rabbit GLUT1 antibodies were added (Table 1). After 1 h, the cells were washed in PBS and the appropriate secondary antibodies anti-mouse Alexa Fluor 488 (A488), (in house) and F(ab')_2_ Donkey anti-Rabbit IgG PE (Affymetrix eBioscience), were added to the cells and allowed to incubate for 1 h. Cells were washed in flow cytometry wash buffer before analysis as stated above. Results of FcγR expression are shown as geometric mean fluorescent intensity (MFI) for FcγR expression on single/live B cells (FSC^−^A^lo^SSC^−^A^lo^CD19^+^CD3^−^), NK cells (FSC^−^A^lo^SSC^−^A^lo^CD3^−^CD56^dim^), monocytes (FSC^−^A^hi^SSC^−^A^lo^CD14^lo/intermediate/high^) and tumor-associated macrophages (FSC-A^hi^SSC^−^A^lo^CD3^−^CD56^−^CD19^−^CD14^−^CD163^+^) (see Additional file 1: Fig. S1a-b for flow cytometric gating strategy for all human immune cells). FcγR, CAIX, CD40, PD-L1 and HLA-DR expression levels were corrected by subtracting the geometric MFI of the corresponding isotype control. Post-staining with fluorophore-conjugated mAbs, cells were analyzed using BD FACSCantoII or FACSCalibur (BD Biosciences) flow cytometers and data analyzed using FlowJo Version 10 software (FlowJo LLC).

**Table 1:** List of primers used for qRT-PCR, to amplify specific regions of the *FCGR2B* promotor, 1 Kbupstream of the TSS

Targeted region	Forward sequence	Reverse sequence
FCGR2B promotor; AP-1 binding site	5'-ATGCTCAATTTCAAGAAGCATCCA-3'	5'-TGAGAAAGGGTGATGCAGGA-3'
FCGR2B promotor; HIF-2α binding site	5'-AGGGAAGGTCCTCACAAGAAT-3'	5'-AGGTTTCGGGTTGAATGCCAG-3'

### Flow cytometry of murine cells

Murine immune cells (from peripheral blood, peritoneum, spleen or bone marrow) were resuspended at 1 × 10^7^ cells per mL in flow cytometry wash buffer. 1 × 10^6^ PBMCs, splenocytes, peritoneal lavage, bone marrow, liver or tumor immune cells in 100 µL were stained with fluorophore-conjugated mAbs per FACS tube, for 30 min at 4 °C. Samples were stained with anti-mouse F4/80 APC (clone: Cl:A3-1, BIO-RAD), anti-mouse Ly6G PE-Cy7 (clone: RB6-8C5, eBioscience), anti-mouse Ly6C PerCP-Cy5.5 (clone: HK1.4, eBioscience) and anti-mouse CD11b Pacific Blue (clone: M1/70, BioLegend). FcγR staining was carried out using anti-mouse FcγRI FITC (clone X54-5/7.1, F(ab') _2_), anti-mouse FcγRII FITC (clone: AT130/2, F(ab')_2_), anti-human FcγRIIb FITC (clone: 6G11, F(ab')_2_), anti-FcγRIII FITC (clone: AT154-2, F(ab')_2_), anti-FcγRIV FITC (clone: AT137, F(ab')_2_), isotype control human IgG1 FITC (clone: FITC8 F(ab')_2_), isotype control mouse IgG2a FITC (clone: 4D5 F(ab')_2_) or isotype control rat IgG2a FITC (clone: Mc106A5 F(ab')_2_) were all generated in-house. Results of FcγR expression are shown as geometric MFI for FcγR expression on single/live macrophages (FSC-A^hi^SSC-A^lo^CD11b^lo^F4/80^+^), monocytes (FSC-A^hi^SSC-A^lo^CD11b^hi^Ly6C^hi^) and neutrophils (FSC-A^hi^SSC-A^hi^CD11b^hi^Ly6G^hi^), see additional file 7: Fig. S7a for flow cytometric gating strategy for murine immune cells. FcγR expression levels were corrected by subtracting the geometric MFI of the corresponding isotype control. Post-staining with fluorophore-conjugated mAbs, cells were analyzed using BD FACSCantoII or FACSCalibur (BD Biosciences) flow cytometers and data analyzed using FlowJo Version 10 software (FlowJo LLC).

### Western blotting

Isolated monocytes (5 × 10^6^) were collected and centrifuged at 800 × *g* for 5 min at room temperature. The supernatant was removed, and the resulting cell pellet was lysed using 20 µL of RIPA buffer (Abcam) supplemented with a Western blot protease inhibitor cocktail (Abcam). The lysed cells were stored at -20 °C. The protein concentration of the lysed cells was determined using a Bradford assay; 50 µg of protein was added to 5 µL of laemmli buffer (Abcam), and distilled H_2_O was added to make each sample a total of 20 µL. The protein-laemmli buffer mix was then heated at 95 °C for 5 min. The samples were loaded onto pre-made 10%, 1.50 mm × 10 well, bis–tris gels (NuPage R) and run at 150 V. Proteins were transferred onto nitrocellulose blotting membranes (GE Healthcare life sciences) in a transfer cassette run at 30 V for 90 min. The proteins probed for were FcγRIIb and HIF-1α and/or HIF-2α and/or JUN with HSC70 as a loading control. The membrane was blocked in a 5% BSA Tris-buffered saline-tween (TBS-T), 0.01% azide solution for one hour. Anti-FcγRIIb (clone:EP888Y, Abcam), anti-HIF-1α (clone: polyclonal, Novus Biologicals) and anti-HSC70 (clone: B-6, Santa Cruz Biotechnology) were added at a 1:500 dilution, anti-HIF2-α (clone: D6T8V), anti-c-Jun (clone: 60A8), anti-phospho-c-Jun (clone: D47G9) and anti-phospho-c-Fos (clone: D82C12, all from Cell Signalling Technology) were added at a 1:1000 dilution. The antibodies were left on the nitrocellulose membrane overnight at 4 °C. The next day, the blots were washed in a 5% BSA Tris-buffered saline-tween (TBS-T) solution followed by a 1 h incubation with horseradish peroxidase (HRP)-linked secondary antibodies. An ECL Western Blotting substrate (Pierce R) was used to detect HRP activity and imaged using the Imager Chemi Doc-It Imaging system (UVP) and the VisionWorks RLS software (UVP). The images were quantified using ImageJ 1.4.3.67 software.

### Oxygen sensing (SensorDish Reader)

PBMCs or purified monocytes were cultured in serum free complete CTL medium in 24-well plates with integrated oxygen sensors (OxoDish-R-DW, PreSens) and placed on the OxoDish R sensor dish reader (SDR) (PreSens) to measure oxygen levels in the culture conditions over a 24 h period, as previously described [41, 42]. The reader and plates were placed at a constant humidity, 37 °C, 5% CO_2_ and oxygen levels were measured every 10 min for 24 h.

### Radiometer analysis of cell culture media

LD and HD human PBMCs or isolated monocytes were cultured for up to 48 h in 24-well plate in serum free CTL medium. Supernatants from these cultures were analyzed for CO_2_, metabolites, pH and Oximetry using an ABL 835 FLEX blood gas analyzer (Radiometer Medical ApS).

### Chromatin immunoprecipitation (ChIP) Assay

ChIP was performed on magnetically sorted isolated monocytes (Pan Monocyte Isolation Kit, human, Miltenyi Biotech), cultured for 10 h at LD with or without 20 µM DMOG, as previously published with several modifications (Hayakawa et al., 2004). Briefly, the SimpleChIP Enzymatic Chromatin IP Kit (Magnetic Beads), (Cell Signalling Technology), was used; 4 × 10^6^ untreated or DMOG treated monocytes were fixed using 37% formaldehyde for 15 min at room temperature. Monocytes were then washed twice in ice cold PBS, centrifuged at 2000 × *g* for 4 min at 4 °C, supernatant discarded and the dried pellets stored at -80 °C overnight. Cell pellets were then thawed on ice and treated with sodium dodecyl sulfate (SDS) containing buffers as per the manufacturer’s protocol. DNA in each sample was digested using 0.5 µL Micrococcal Nuclease per immunoprecipitation (IP) and incubated for 15 min at 37 °C. The digestion was stopped using 0.5 M ethylenediaminetetraacetic acid (EDTA) and after further washing and treatment with buffers as per the manufacturer’s protocol, the digests sonicated using a Soniprep 150 sonicator (MSE), at setting 3 for 15 cycles of 45 s on and 15 s off, whilst being kept on ice. The lysates were then clarified by centrifugation at 9,400 × g for 10 min at 4 °C. The supernatants were removed and stored at -80 °C overnight. 50 µL of this sample was run on a 1% agarose gel following digestion with RNAse A and Proteinase K, as per the manufacturers protocol using the SimpleChIP Enzymatic Chromatin IP Kit, (Cell Signalling Technology). DNA for all samples was observed to be fragmented between 150–900 bp. Each DNA sample was then incubated overnight at 4 °C with Rabbit mAb IgG XP isotype control (clone DA1E) for the negative control IP, Histone H3 XP Rabbit mAb (clone: D2B12) for the positive control, c-Jun Rabbit mAb (clone: 60A8), HIF-1α XP Rabbit mAb (clone D1S7W) or HIF-2α Rabbit mAb (clone: D6T8V, all mAbs purchased from Cell Signalling Technology). The chromatin from each sample was then separated from the aforementioned mAbs using protein G magnetic beads followed by reversal of chromatin/DNA cross linking and DNA purification as per the manufacturer’s protocol. Using real-time quantitative polymerase chain reaction (RT-QPCR) *RPL30*; the positive control gene was amplified from DNA isolated via the anti-Histone H3 ChIP assay. Commercially available primers (Cell Signalling Technology) and the SimpleChIP Universal qPCR Master Mix (Cell signalling Technology) were then used to detect *RPL30* as per the manufacturer’s protocol. Using the same SimpleChIP Universal qPCR Master Mix the *FCGR2B* promotor region was also amplified at specific regions predicted to contain AP-1 or HIF-α binding motifs within the 1 Kb gene promotor upstream of the transcription start site (TSS), using custom-designed primers (see Additional file 1: Table S1 for list of primers, purchased from Integrated DNA Technologies).

### Whole genome DASL (cDNA-mediated annealing, selection, extension and ligation) Array and bioinformatic analyses

Monocytes were isolated from PBMC cultures (using the Pan Monocyte Isolation Kit, human, Miltenyi Biotech), cultured at HD, and harvested at 0, 2, 10 and 24 h from 2 donors. B cells were also isolated from PBMC cultures (using the B cell Isolation Kit II, human, Miltenyi Biotech), cultured at HD and harvested at 0 and 24 h. Total RNA was isolated from these cells using the RNAeasy Mini Kit (Qiagen) for assessment on the whole genome DASL array.

A whole genome DASL array was carried out on monocyte and B cell total RNA samples. The resulting dataset was corrected for background using negative controls and normalized using the neqc function of the limma v3.24.15 Bioconductor package [43] in R v3.3.2 (R Core Team (2017). R: A language and environment for statistical computing. R Foundation for Statistical Computing, Vienna, Austria. URL https://www.R-project.org/). The dataset was also quality checked by only including probes that were expressed in at least 3 arrays according to detection *p*-values of 5%. Multi-dimensional scaling plots were generated using the plotMDS function to look at the variability between donors and time points. The normalized, quality checked data was then used to evaluate differentially expressed genes between the 4 time points and a cut-off based on a defined FDR was used to generate a list of gene candidates between every iteration of comparisons. These identified genes were used to generate heatmaps in Ingenuity Pathway Analysis (IPA) v01-07. Gene set enrichment analysis was performed using the fgsea R package (Korotkevich, 2019, https://doi.org/10.1101/060012) and referenced to Broad hallmark gene sets (h.all.v7.2). Genes were pre-ranked using a signed log_10_-transformed FDR from differential analysis in DESeq2 (Love, 2014), with the sign denoting the direction of logFC.

### RNA-Seq

Monocytes were isolated from PBMCs using the Pan Monocyte Isolation Kit, human, (Miltenyi Biotech), from 7 different healthy adult donors and cultured at LD (1 × 10^6^/mL in a 24-well plate) in the presence or absence of 20 μM DMOG, in complete CTL medium at 37 °C and 5% CO_2_. Complete CTL medium was removed from monocyte cultures and they were disrupted using QIAzol lysis reagent (Qiagen) and total RNA was then isolated using the miRNeasy mini kit (Qiagen) as per the manufacturer’s protocol at 0, 2, 10 and 24 h post-culture. RNA quantity and quality for each sample was assessed using the RNA 6000 Nano kit (Agilent), analyzed using a Bioanalyzer (Agilent) and only samples with RIN scores > 8 were used for further downstream analysis. RNA samples were enriched for mature, poly-A mRNA transcripts and 150 base pair paired-end sequencing was carried out on the NovaSeq 6000 (Illumina) platform by Oxford Genomics Centre (Oxford, UK) resulting in an average of 39 million reads per sample. Sequencing reads were aligned to the human genome (primary assembly, GRCh38.p12, Ensembl) [44, 45] using the STAR alignment algorithm [46] and uniquely-mapped alignments overlapping gene exons were counted using featureCounts from the Rsubread package [47]. Counting was performed relative to Ensembl 97 (Jul 2019) gene annotation and counts summarized at the gene level. Gene expression filtering and normalization was carried out in R, using edgeR [48]. Genes with below-threshold counts were filtered out (filterByExpr: min.count = 30, min.count.total = 45) and between-sample normalization was performed using the trimmed mean of M-values method. Gene expression is reported as counts-per-million (CPM).

### RNA-Seq differential gene expression

Differential expression analysis was carried out using the limma [43] linear-modelling R package and specifically the voomWithQualityWeights function to provide gene- and sample-specific weights to account for mean–variance relationships in the data. A group-means approach was taken for the design matrix, with donor as a blocking variable. Between-treatment comparisons were made at 2, 10, and 24 h for DMOG versus untreated and across-time comparisons were also made for the following between-treatment comparisons; 10 h versus 2 h and 24 h versus 10 h. Differences in expression across-time were also assessed within-treatment for DMOG and untreated separately; 2 h versus 0 h, 10 h versus 2 h and 24 h versus 10 h. Differential expression tests were performed for each comparison with a null interval hypothesis for the expression fold change (FC) [-log2(1.2) < log2(FC) < log2(1.2)] with false discovery rate (FDR) < 0.05 per comparison using the Benjamini–Hochberg procedure. Principal Component Analysis was performed using genes that are differentially expressed (in either direction) in at least one of the between-treatment comparisons.

### ATAC-seq (assay for transposase-accessible chromatin using sequencing)

ATAC-seq was performed as previously described [49–51], with minor alterations. Monocytes sourced from healthy adult donors were isolated from PBMCs using the Pan Monocyte Isolation Kit, human, (Miltenyi Biotech) and cultured in complete CTL medium at 37 °C in 5% CO_2_. For the experiment comparing LD and HD culture conditions, monocytes from 3 donors were either plated at 1 × 10^6^ cells/mL (LD) or 1 × 10^7^ cells/mL (HD) and cultured for 24 h. For the time course experiment assessing DMOG treatment, monocytes from 7 donors were cultured at 1 × 10^6^ cells/mL either with or without 20 µM DMOG for 24 h. ATAC-seq library preparation was the same in both cases: 50,000 monocytes per sample were harvested at 24 h post-culture and centrifuged at 300 × *g* for 5 min at 4 °C. The cell pellet was carefully resuspended in transposase reaction mix (12.5 μL 2 × TD buffer, 2 μL TDE1 (Illumina)), 10.25 μL nuclease-free water and 0.25 μl 1% digitonin (Sigma-Aldrich) per sample, for 30 min at 37 °C. 11 μL of DNA was isolated from each sample using the MiniElute PCR Purification Kit (Qiagen). 1 μL of eluted DNA from each sample was used in a quantitative PCR (qPCR) reaction to estimate the optimum number of amplification cycles. The remaining 10 μL of each library was amplified for the number of cycles corresponding to the C_q_ value from the qPCR (the cycle number at which fluorescence has increased above background levels). Library amplification was followed by Solid Phase Reversible Immobilization (SPRI, Beckman Coulter) size selection to exclude fragments > 1,200 bp. DNA concentration was measured with a Qubit fluorometer (Life Technologies) and library amplification was performed using custom Nextera primers [49]. Libraries were sequenced by the Biomedical Sequencing Facility at CeMM (Vienna, Austria) using the Illumina HiSeq 3000/4000 platform. 50 bp single-end sequencing was performed for the LD-HD comparison experiment with two technical replicate sequencing runs per sample library. 75 bp paired-end sequencing was performed for the DMOG time course experiment.

### ATAC-Seq data analysis

For the LD-HD comparison experiment, sequencing reads were aligned to the human genome (GRCh38) using HISAT2 v2.1.0 [52] and the non-default parameter –no-spliced-alignment. Peaks were called for each sample and technical replicate separately using MACS2 v2.2.1 [53], callpeak function with non-default parameters –nomodel –shift -100 –extsize 200 -B –broad. For the DMOG time course experiment, sequencing reads were trimmed of adapter sequences using cutadapt v2.4 (Marcel Martin, 2020, https://doi.org/10.14806/ej.17.1.200) and aligned to the human genome (GRCh38) using HISAT2 v2.1.0 and the non-default parameter –no-spliced-alignment. Peaks were called for each sample separately using MACS2 v2.2.6 callpeak function with non-default parameters –nomodel –call-summits. For both experiments, read counting in peaks was performed in R with the diffbind package for differential open region calling [54], using only uniquely-mapped reads and the DEseq2 option [55]. Peak annotation was performed using the ChIPseeker package in R [56] and GENCODE 32 annotation [57].

For the DMOG time course experiment transcription factor (TF) binding sites that overlapped peaks were determined using the Open Regulatory Annotation database (ORegAnno) 3.0 [58] and the bedtools function closest [59]. Differentially open peaks between DMOG-treated or untreated monocytes were scanned for TF binding sites and the occurrence frequency of each TF over all differentially open peaks was calculated. To determine if these frequencies were significant or obtained randomly, the same number of regions as differentially open peaks were selected randomly from the genome and scanned for the same TFs. This process was repeated 1000 times to a generate a per-TF frequency distribution for randomly selected regions. Z-scores were calculated for the observed TF frequency in differentially open regions with respect to the random region frequency distribution.

### Chromatin immunoprecipitation-Seq data analysis

Publicly available FASTQ files for samples from Tausendschon et al. [60], (GSE43109), were aligned to the human genome hg19 using bowtie (v1.1.2, pre-built hg19 bowtie index: https://benlangmead.github.io/aws-indexes/bowtie) [61], with the following alignment reporting parameters; -k 4 -m 4 –best. This allows for multi-mapping alignments which occur in the *FCGR* low affinity locus due to sequence homology. The multi-mapping alignment positions were checked for those reads that aligned to peaks approximately 10 Kb upstream of *FCGR2B* and *FCGR2C* transcriptional start sites. These reads only multi-map between homologous sequences of *FCGR2B* and *FCGR2C* and not elsewhere on the genome.

### RBC phagocytosis assay

Whole blood samples were sourced from Rhesus D positive healthy adult female donors. Monocytes were also isolated from these samples using density centrifugation (Lymphoprep) and the Pan Monocyte Isolation Kit, human (Miltenyi Biotech). Monocytes were then cultured in complete RPMI for 48 h at LD and HD as previously described. Whole blood samples were taken again from the same Rhesus D positive donors and RBCs isolated using density centrifugation (Lymphoprep). RBCs were then were labelled with 2 µM Carboxyfluorescein succinimidyl ester (CFSE), quenched with complete RPMI and washed twice in complete RPMI. These RBCs were then opsonized with 5 ug/mL anti-Rhesus D hIgG1 antibody or cetuximab isotype control in flow cytometry wash buffer and washed once. 5 × 10^5^ CFSE labelled and antibody pre-treated RBCs were cultured with 1 × 10^5^ autologous LD or HD monocytes (5:1 target:effector ratio) in 100 uL complete RPMI per well, in a 96-well plate. Alternatively, mAb-opsonized and CFSE-labelled RBCs were cultured with autologous LD or HD monocytes pre-treated with E08 (anti-human-FcγRIIa) or 6G11 (anti-FcγRIIb) F(ab’)_2_ fragments to block IgG Fc-FcγRII interactions. After a 2 h incubation period at 37 °C, and 5% CO_2_, the monocytes were stained with anti-CD14 Pacific Blue (Biolegend), washed once, and CD14^+^CFSE^+^ (Monocytes that had phagocytosed RBCs) of total CD14^+^ cells were quantified using a BD FACSCantoII (BD Biosciences) flow cytometer and data analyzed using FlowJo Version 10 software (FlowJo LLC).

### MDM antibody-dependent cellular phagocytosis (ADCP) assay

MDM phagocytic function was assessed as reported previously [62]. In brief, M0, M1 or M2 macrophages were seeded at 1 × 10^5^ cells per well in a flat bottomed 96-well plate in 100 µL of complete RPMI. CLL cells were used as targets and labelled with 5 µM CFSE for 10 min and washed with complete RPMI. CLL cells were opsonized with Herceptin or cetuximab (negative control) and rituximab or obinutuzumab, incubated at 37 °C and 5% CO_2_ for 30 min. Opsonized target cells were then washed and added to the MDMs at an effector to target ratio of 1:5, incubated at 37 °C and 5% CO_2_ for 2 h. Cells in each well were labelled with anti-human FcγRIIIA-APC (BioLegend), with target uptake determined using the BD FACSCantoII or FACSCalibur (BD Biosciences) flow cytometers and analyzed using FlowJo Version 10 software (FlowJo LLC).

### In vivo studies

WT C57BL/6 J or BALB/c mice were inoculated with MCA205, EG7 or CT26 tumor cells (5 × 10_5_) subcutaneously (s.c.) into the right flank and mice sacrificed once tumors sizes reached 500 mm^3^. Tumors and spleens were then harvested and FcγR expression on monocytes and macrophages in these tissues determined using flow cytometry.

Additionally, WT C57BL/6 J mice were inoculated with E0771 tumor cells (2.5 × 10^5^, injected into mammary fat pad) and mice sacrificed once tumors sizes reached 500 mm^3^. To determine hypoxic regions within these tumors, Hypoxyprobe (pimonidazole), Hypoxyprobe-RedAPC Kit (Hydroxyprobe™) was resuspended at a concentration of 30 mg/mL in 0.9% sterile saline. Multiple mice (*n* = 5 per tumor type) were injected intravenously (i.v., tail vein) with 60 mg/kg of the pimonidazole solution. Mice were sacrificed 90 min later, tumors harvested, embedded in OCT (CellPath, Newtown, Powys, U.K.) and frozen in isopentane on a bed of dry ice. Tumor samples were stored in plastic scintillation vials at − 80 °C [63]. In order to assess the effects of HIF-PH inhibitor treatment on FcγR expression on myeloid cells in vivo, gender- and age-matched WT C57BL/6 J mice were dosed with 4 mg DMOG or PBS vehicle control via intraperitoneal (i.p.) route on three consecutive days. Mice were sacrificed 24 h later, the blood and peritoneal lavage harvested and FcγR expression levels assessed on monocytes, neutrophils and macrophages, using flow cytometry.

WT C57BL/6 J mice were also dosed with 200 µg Roxadustat or PBS control i.p. on three consecutive days. Mice were sacrificed 24 h later, the blood, peritoneal lavage, spleen and bone marrow were harvested and FcγR expression levels assessed on neutrophils, monocytes and macrophages in each compartment, using flow cytometry.

In order to assess whether HIF-PH inhibitors can impair mAb-mediated target cell depletion in vivo, age- and gender-matched WT C57BL/6 J mice were treated with 200 µg Roxadustat or PBS i.p. on two consecutive days. 20 h later 10 µg of anti-mouse CD20 mAb; 18B12 or isotype control mAb (DB7/12) was given i.v. Mice were treated again with 200 µg of Roxadustat or PBS i.p. before peripheral blood was taken (tail bleed) to assess systemic levels of CD19^+^ cells 24 h later, using flow cytometry. In separate experiments, age- and gender-matched WT C57BL/6 J mice were dosed with 200 µg Roxadustat or PBS i.p. on two consecutive days prior to receiving CFSE labelled EL4-huCD20^+^ cells i.p. on the second day. On the third day mice were treated with 50 ug of rituximab or cetuximab i.v. followed by a final treatment with 200 µg of Roxadustat or PBS also on the third day. EL4-hCD20^+^ cell depletion in the peritoneal lavage following mAb treatment was quantified using flow cytometry 24 h later.

In order to assess the effects of HIF-PH inhibitor treatment on human FcγRIIb expression on myeloid cells and systemic B cell depletion in vivo, Tg hFcγRIIb^+/-^ x mFcγRII^−/−^ x hCD20^+/-^ mice were dosed with 4 mg of DMOG or PBS vehicle control i.p. on two consecutive days. Mice were treated with 50 µg rituximab or cetuximab i.v. followed by a final treatment with 4 mg DMOG or PBS also on the third day. Mice were sacrificed 24 h later with peritoneal lavage, spleen, and bone marrow harvested. FcγR expression levels were assessed on monocytes, neutrophils, and macrophages and frequencies of live B (CD19^+^) cells quantified using flow cytometry.

In order to assess the effects of HIF-PH inhibitor treatment on specific hCD20^+^ B cell depletion in vivo, an adoptive transfer assay was performed as before [30]: Tg hFcγRIIb^+/−^ x mFcγRII^−/−^ mice were dosed with 4 mg of DMOG or PBS vehicle control i.p. on three consecutive days. On the third day mice were given 3 × 10^6^ target splenocytes from hCD20^+/−^ x mFcγRII^−/−^ mice and 3 × 10^6^ non-target splenocytes from WT C57BL/6 J mice differentially labelled with CFSE, i.v. These mice were again treated with 4 mg of cetuximab or PBS i.p. prior to receiving 50 µg rituximab or cetuximab 24 h later. Depletion of target and non-target splenocytes in the spleen was quantified using flow cytometry. Finally, Tg hFcγRIIb^+/−^ x mFcγRII^−/−^ mice were treated with 4 mg DMOG or PBS i.p. on three consecutive days, prior to receiving CFSE labelled EL4-huCD20^+^ cells i.p. on the third day. These mice were then treated with 50 µg Rituximab or Cetuximab i.v. followed by a final treatment with 4 mg DMOG or PBS on the fourth day. EL4-huCD20^+^ cell depletion in the peritoneal lavage following mAb treatment was quantified using flow cytometry 24 h later.

### Immunofluorescence microscopy

Fresh murine spleen and CT26, MC38 or E0771 tumor tissue were embedded in OCT (CellPath, Newtown, Powys, U.K.) and frozen in isopentane on a bed of dry ice. Sections (8 μm) were cut, air-dried (overnight), fixed in 100% acetone for 10 min and blocked with 2.5% normal goat serum before incubation with anti-mouse FcγRII (clone: AT130-2, in house). Murine FcγRII was detected (45 min) with Alexa Fluor 488–conjugated anti-rat IgG (Life Technologies)). When using a second rat primary antibody, sections were incubated with rat IgG (50 μg/mL, 30 min, prepared in house). Hypoxic regions within tumor sections were detected by staining for pimonidazole using the Hypoxyprobe-RedAPC Kit (1000 mg pimonidazole HCl plus 2 units of 4.3.11.3 mouse Dylight™APC-Mab, Hydroxyprobe™) as per the manufacturer’s protocol. Sections were mounted in Vectashield Hardset (Vector Laboratories). Images were collected using a CKX41 inverted microscope with reflected fluorescence system equipped with a CC12 color camera running under Cell B software, using Plan Achromat 10 × 0.25 and 40 × 0.65 objective lenses (all from Olympus, Southend-on-Sea, Essex, U.K.). RGB image files (.tif) were transferred to Adobe Photoshop (CS6; Adobe Systems, San Jose, CA) and all images treated in the same way. Tissue autofluorescence was removed by difference blending between the color channels and red/green image overlays contrast-stretched to use the whole grayscale. Colocalization analysis was performed using the Coloc2 plugin (https://imagej.net/plugins/coloc-2) in Fiji [64]. Background was measured in unstained areas of the section and the mean plus 2 times standard deviation subtracted from the image. Coloc2 was set to measure Manders coefficients using Costes threshold regression and a PSF of 3 and to perform a Costes significance test with 10 randomisations (Costes *P* value > 0.95 denotes non-random colocalization).

### Statistical analysis

Statistical significance when comparing FcγR expression in vitro on human immune cells on untreated versus treated cells or between groups of untreated and HIF-PHD inhibitor treated mice was determined between the groups using either an unpaired two-tailed t-test or paired two-tailed Wilcoxon tests. One-way analysis of variance (ANOVA) was used with the Bonferroni correction for multiple comparisons as needed, to compare multiple treatment groups when assessing mAb mediated cell depletion in vivo. As a large number of statistical tests have been carried out in a range of contexts, *p*-values should be interpreted with care and within the overall scientific context. Data analysis was carried out using the Graphpad Prism version 8.0.1 software. Statistical significance defined as ^*^p < 0.05, ^**^p < 0.01 ^***^p < 0.001 and ^****^p < 0.0001 and ns = non-significant.

## Results

### High density cell culture elicits marked induction of FcγRIIb expression on human monocytes

Leukocyte-based in vitro assays remain one of the gold standards for determining mAb efficacy and predicting adverse responses in patients [65]. We have previously characterized several assay formats reporting on the impact of FcγRs on mAb mediated immune cell responses in vitro [32, 66]. This work led us to observe that when human primary monocytes are cultured at high density (HD; ~ 8 × 10^6^ cells per cm^2^) they markedly upregulate the inhibitory Fc receptor for IgG; FcγRIIb [32]. FcγRIIb impairs myeloid cell effector functions by antagonizing activating FcγRs and so we sought to further understand how high cell density modulates FcγR gene and protein expression. Concordant with our previous findings [32] the high affinity activating IgG receptor, FcγRI, remained unaltered under HD conditions, whereas the expression of the low affinity FcγRs; FcγRIIa, FcγRIIb and FcγRIIIa were all significantly increased on monocytes in HD PBMC cultures relative to monocytes from LD cultures (LD; ~ 0.8 × 10^6^ cells per cm^2^), 48 h post-culture (*p* < 0.0001 for all low affinity FcγR comparing LD versus HD monocytes). Although, there were significant increases in FcγRIIa (~ twofold) and FcγRIIIa (sevenfold), HD culture induced a striking ~ 110-fold increase in FcγRIIb expression on monocytes (Fig. 1a). Relative quantification of FcγR expression using PE-conjugated beads, allowed us to ascertain that HD monocytes had a tenfold lower activating to inhibitory (A:I) FcγR expression ratio in comparison to LD monocytes (Fig. 1b). The full extent of the profound FcγRIIb upregulation was highlighted when we compared its expression to other monocyte cell surface markers, including MHC class I/II molecules, CD33, CD11b, CD14, CD83 and CD86, showing it to be significantly higher (*p* < 0.01, comparing FcγRIIb versus MHC class I expression levels, Fig. 1c). Western blotting confirmed that both FcγRIIb1 and FcγRIIb2 isoforms [67] were upregulated with FcγRIIb2 the dominant isoform expressed (Fig. 1d-e). Upregulation of FcγRIIb on HD monocytes was confirmed using multiple anti-FcγRIIb and pan FcγRII specific mAbs (Additional file 1: Fig. S1c). When comparing LD versus HD cultures, there was no significant difference in the expression of FcγRIIb on B cells, nor FcγRIIIa on NK cells, which was confirmed by flow cytometry PE bead quantification, demonstrating that FcγR expression on these cell subsets remained unaffected under HD culture (Additional file 1: Fig. S1d). CD3^+^ T cells remained FcγR negative regardless of cellular density (data not shown). In conclusion, we observed that although all low affinity FcγRs were upregulated on monocytes, this was not observed on other cell types within the same PBMC culture and the most striking feature was the marked and specific upregulation of the inhibitory FcγRIIb on the monocytic population.

### High cell density elicits a hypoxia related gene signature and metabolic perturbation in human monocytes

**Fig. 2:**
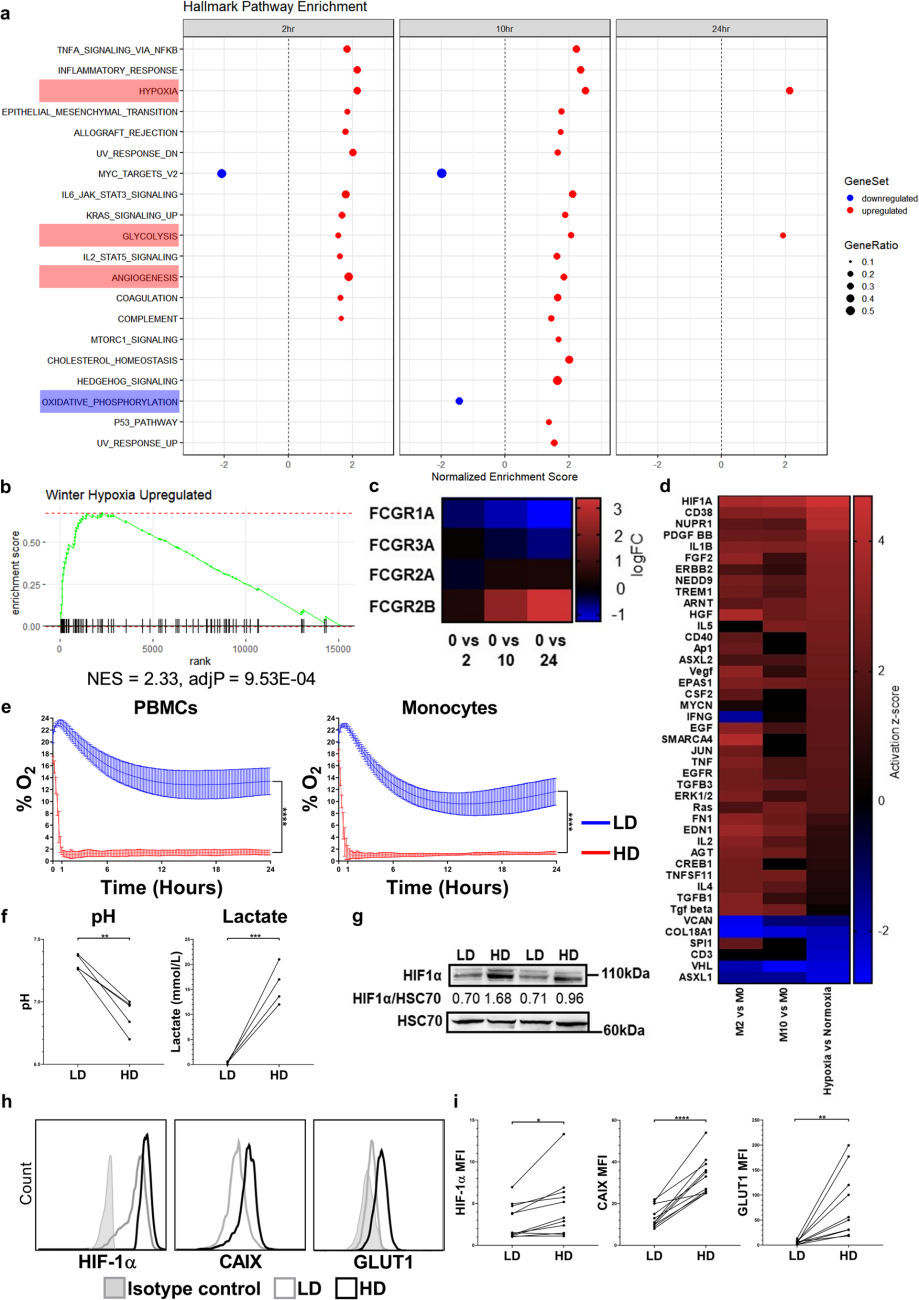
Transcriptional and physiological profiling of HD human monocytes. The transcriptome of fresh and HD human monocytes cultured for 2, 10 or 24 h was investigated using microarray analysis. **a**, Pre-ranked GSEA; genes were ranked according to their differential expression between monocytes at 2, 10 or 24 h post-HD culture and fresh monocytes. Twenty Hallmark gene sets (v7.2) were significantly overrepresented (FDR < 0.05). Upregulated gene expression is signified in red and downregulation in blue. **b**, Enrichment plot of the Winter hypoxia gene set in monocytes post-HD culture. **c**, Heat map of *FCGR* gene expression. **d**, Microarray gene expression data was acquired using fresh monocytes (M0) and monocytes at 2 (M2),10 (M10) and 24 (M24) hours post-HD culture as well as monocytes cultured under hypoxic conditions (1% O_2_) for 24 h (Bosco et al., 2006). Heat map of activation z-scores for the top 50 genes and proteins determined to be the most activating or inhibiting. **e**, % O_2_ in LD and HD cultures of human PBMCs and isolated monocytes (*n* = 6 per group, thickness of lines for LD and HD represent SEMs for each time point). **f**, pH and Lactate in donor matched LD and HD PBMC culture supernatants (*n* = 5 per group). **g**, LD and HD monocyte cell lysates were generated and HIF-1*α* and HSC70 expression assessed using Western blotting. Representative Western blot staining for 2 donors is shown. **h-i**, Representative histograms and graphs showing expression of HIF-1α, CAIX and GLUT1 expression quantified using flow cytometry of LD and HD precultured monocytes (*n* = 11 per group). Each point on the graphs represents a unique healthy donor. Statistical significance between groups was assessed by using a paired two-tailed Wilcoxon test (*p < 0.05, **p < 0.001, ***p < 0.001 and ****p < 0.0001). Also see Additional file 2: Fig. S2

Next, we performed a transcriptomic characterization of HD monocytes to investigate gene expression changes which were associated with the enhancement of monocyte FcγRIIb expression. A whole genome DASL array was carried out on donor matched samples of fresh purified peripheral blood monocytes (M0) versus monocytes cultured at HD for 2 h (M2), 10 h (M10) and 24 h (M24), alongside fresh purified human B cells (B0), and purified B cells cultured at HD for 24 h (B24), sourced from two healthy human donors. Multidimensional scaling plots of gene expression revealed considerable divergence between fresh monocytes versus 24 h cultured HD monocyte samples, with this observation being mirrored by fresh B cells versus 24 h cultured B cell samples, indicating marked global gene expression changes occurring under HD culture in both immune cell subsets (Additional file 2: Fig. S2a). Gene set enrichment analysis (GSEA) was performed to assess the biological processes associated with these differentially expressed genes. The top 20 statistically significant Hallmark pathway enrichment categories revealed a prominence of stress, inflammatory and, to a greater extent, hypoxia related processes in HD monocyte cultures. We observed a downregulation of the oxidative phosphorylation related gene expression signature and conversely an upregulation of hypoxia and glycolysis related gene expression in HD monocytes after 10 and 24 h (Fig. 2a). Owing to the prominence and importance of these changes in the TME, we chose to focus further on these aspects. GSEA confirmed that even within 2 h, gene expression in HD monocyte cultures was enriched for the Winter Hypoxia Metagene gene set (NES = 2.02 and FDR = 0, Fig. 2b) – a gene set involved in the hypoxia pathway in the TME [68]. Gene expression analysis also confirmed the upregulation of FcγRIIa, FcγRIIIa and particularly FcγRIIb on HD monocytes when compared to fresh monocytes (Fig. 2c). Ingenuity pathway analysis (IPA) of differential gene expression revealed that several genes associated with the hypoxia pathway, such as *HIF1A*, *HIF2A* (*EPAS1*), and *ARN*T (*HIF1β*) were amongst the top 50 upstream regulator genes and proteins in HD monocytes. In addition *JUN*, encoding c-Jun, a protein which forms the AP-1 transcription factor complex alongside c-Fos and which was previously reported to regulate *FCGR2B* gene expression [69] was also a prominent upstream gene regulator. Comparing the differentially expressed genes in HD monocytes with differentially expressed genes in hypoxic monocytes (monocytes cultured at 1% O_2_) versus normoxic monocytes generated in a previous study [70], revealed high similarity in the upstream regulators of differentially expressed genes in HD monocytes and hypoxic monocytes (Fig. 2d). Interestingly, HIFs were significantly associated with upstream gene regulation in HD monocytes when compared to B cells at 24 h post-culture. Furthermore, AP-1 or its constituent proteins did not feature amongst the top 50 most differentially expressed upstream regulators of gene expression in the monocyte versus B cell comparisons, indicating differential transcriptional responses to HD culture in the two immune cell types (Additional file 2: Fig. S2b). To directly confirm whether HD cultures of PBMCs and monocytes were indeed hypoxic, we measured % O_2_ levels using an SDR SensorDish® Reader and observed that O_2_ levels dropped from 21 to 1% within 90 min in HD cell cultures, whereas in LD cell cultures O_2_ levels remained ≥ 12% even after 24 h (Fig. 2e). We also observed a significant reduction in pH and corresponding increases in lactate levels in HD PBMC culture supernatants (Fig. 2f), as well as significant reductions in acid base excess and corresponding reductions in HCO_3_^−^(P), (Additional file 2: Fig. S2c-d). Although glucose levels significantly decreased in HD cultures at 48 h, glucose levels remained high (> 9 mmol/L, at pre-diabetic levels [71]) in the culture media (Additional file 2: Fig. S2e). Electrolyte levels were not significantly altered when comparing HD versus LD culture supernatants (Additional file 2: Fig. S2f-i). To further validate the rapid emergence of hypoxia in HD monocyte cultures, we measured expression levels of HIF-1α, the master transcriptional regulator of cellular hypoxia [72], by Western blotting and confirmed enhanced HIF-1α protein accumulation in HD monocytes (Fig. 2g and Additional file 2: Fig. S2j-k). Using flow cytometry, moderate but significant increases in HIF-1α expression levels were confirmed in HD monocytes relative to LD monocytes (*p* < 0.05). Furthermore, two HIF-1α target genes; Glucose transporter 1 (GLUT1) and Carbonic anhydrase IX (CAIX) were also significantly upregulated on HD monocytes (*p* < 0.001 and *p* < 0.0001, respectively; Fig. 2h-i). The latter two genes are established indicators of hypoxia, and ATAC-Seq analysis of HD monocytes further revealed increased openness of not only the *HIF1A* gene itself but additionally HIF responsive genes *ENO2*, *GLUT1,* and *CXCR4* (Fig. S2i). These observations led us to hypothesize that hypoxia upregulates FcγRIIb expression in mononuclear phagocytes. Therefore, we next applied an immunophenotyping and integrative multi-OMIC approach to investigate the mechanism by which HIF activation may enhance FcγRIIb expression on mononuclear phagocytes.

### HIF-prolyl hydroxylase (HIF-PH) inhibition induces FcγRIIb upregulation on human monocytes

**Fig. 3:**
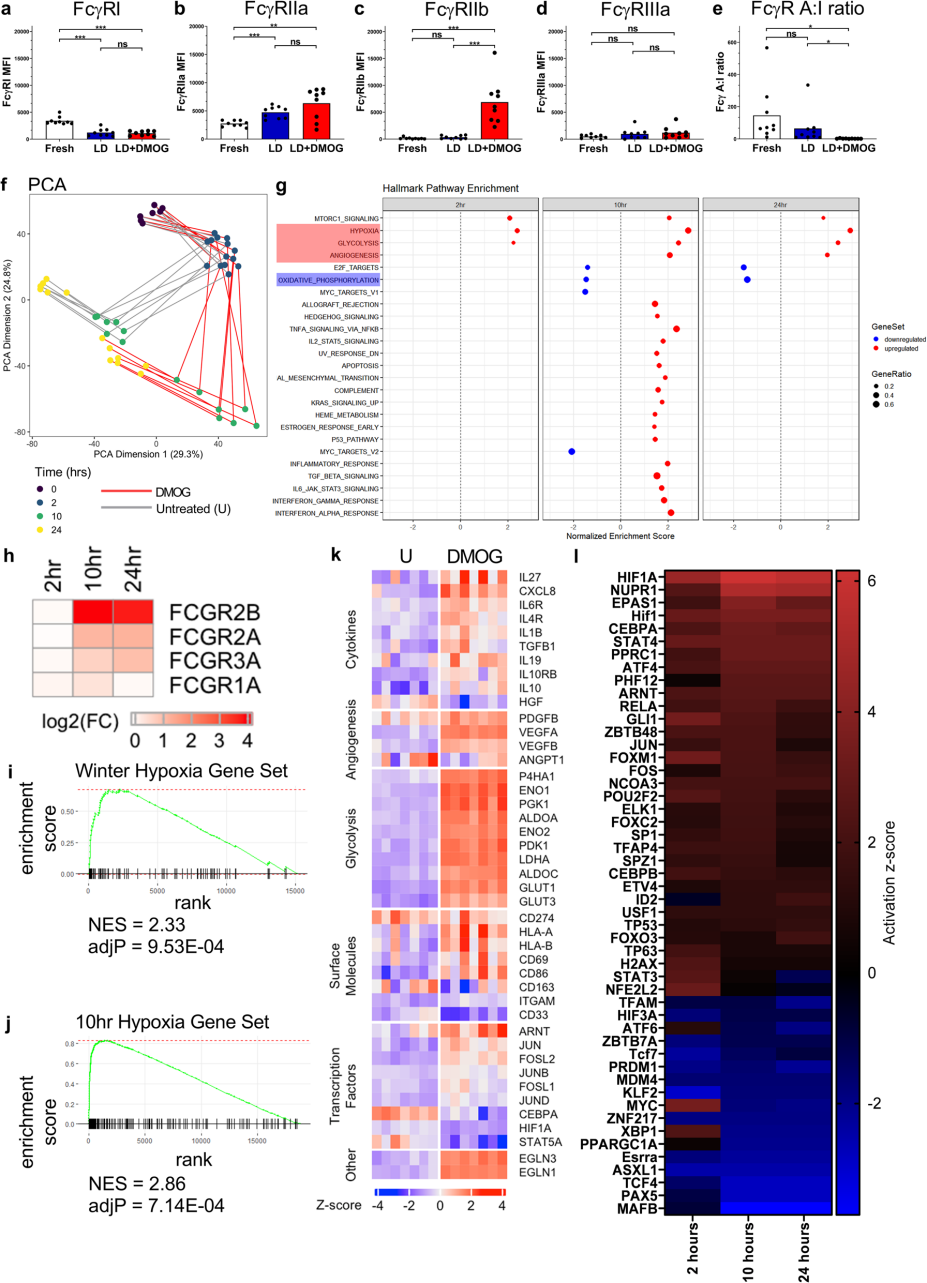
Transcriptional profiling and immunophenotyping of human monocytes during HIF-prolyl hydroxylase inhibition. **a**-**e**, FcγR expression levels and FcγR A:I (FcγR activating:inhibitory) ratio were quantified using flow cytometry (*n* = 7). Statistical significance between groups was assessed using a paired two-tailed Wilcoxon test (*p < 0.05, **p < 0.01 and ***p < 0.001). **f**-**l**, RNA-Seq analysis of the transcriptome of untreated and DMOG treated monocytes cultured for 0 (fresh), 2, 10, and 24 h. **f**, Monocyte gene expression time course trajectories in principal component space (dimensions 1 and 2). Principal Component Analysis (PCA) on 6198 differentially expressed genes for untreated versus DMOG-treated comparisons. **g**, Pre-ranked GSEA; genes were ranked according to their differential expression between monocytes at 10 h post-DMOG treatment and fresh monocytes. Twenty-five Hallmark gene sets (v7.2) were significantly overrepresented (FDR < 0.05), with gene sets of interest highlighted red, indicating upregulated gene expression across all time-points, and the oxidative phosphorylation gene set highlighted blue, showing downregulation of gene expression. **h** Expression fold changes (log2(FC)) for *FCGR* genes between untreated and DMOG-treated monocytes. **i**, Enrichment plot of Winter hypoxia gene set in monocytes at 10 h post-DMOG treatment vs fresh monocytes (NES = 2.33). **j**, Enrichment plot of the Hallmark hypoxia gene set in DMOG treated monocytes at 10 h vs fresh monocytes (NES = 2.86). **k**, Gene expression heat map for differentially expressed genes of interest in untreated (U) and DMOG-treated monocytes at 10 h post-culture. Columns represent monocyte samples from 7 donors. **l**, Upstream regulator analysis was performed using IPA. Heat map of activation z-scores indicates the top 50 transcription factor genes and proteins predicted to be activating or inhibiting when comparing untreated monocytes with DMOG treated monocytes (*n* = 7 per group). Also see Additional file 3: Fig. S3

HIFs are constitutively expressed in all cells, however, in the presence of molecular O_2_ they are rapidly degraded through the action of HIF-PH enzymes. In the absence of oxygen; i.e. hypoxia, HIF-PH-mediated hydroxylation of HIF-α subunits and their subsequent degradation is inhibited, allowing HIF-α to accumulate, facilitating its dimerization with HIF-1β (ARNT) and subsequent target gene binding and transcription [73]. Endogenous HIF protein levels can be increased by the suppression of HIF-PH activity using small molecule competitive inhibitors of the HIF-PHs such as Dimethyloxalylglycine (DMOG) [74, 75]. Several studies have used DMOG as a hypoxia-mimetic in mouse models of inflammation and LPS induced septic shock to alleviate pathology [76–79]. We utilized DMOG for its previously reported ability to activate HIFs and induce downstream gene expression patterns that show concordance with those observed under physiological hypoxia [80]. Peripheral blood monocytes from 7 adult healthy donors were sampled at 0 h (freshly isolated) or after 2, 10 or 24 h, post-culture with and without DMOG. Immunophenotyping using flow cytometry 24 h post-culture showed that DMOG treatment of LD monocytes resulted in a significant decrease in FcγRI expression (*p* < 0.001, Fig. 3a), significant increase in FcγRIIa expression (*p* < 0.01, Fig. 3b) and non-significant change in FcγRIIIa expression (Fig. 3d). However, as observed under HD conditions the most marked change was the pronounced enhancement of FcγRIIb expression in response to DMOG treatment (*p* < 0.001, Fig. 3c). Consequently, the FcγR A:I ratio was significantly decreased in DMOG treated monocytes (*p* < 0.05, Fig. 3e). RNA-Seq analysis was then carried out on these 7 healthy donor monocyte samples across the 24-h time-frame. Monocyte gene expression time course trajectories visualized by PCA using data from 6198 differentially expressed genes when comparing untreated versus DMOG treated monocytes, revealed considerable divergence at the 10 and 24 h time points between untreated versus treated samples, indicating large changes in the transcriptome of DMOG treated monocytes (Fig. 3f). GSEA was performed to assess the biological processes associated with differentially expressed genes. The top 20 statistically significant Hallmark pathway enrichment categories revealed a prominence of stress, inflammatory and hypoxia related processes in DMOG treated monocytes (Fig. 3g). Importantly, we observed a downregulation of the oxidative phosphorylation gene set and conversely an upregulation of hypoxia, angiogenesis and glycolysis gene sets, in 10 and 24 h cultured DMOG treated monocytes (Fig. 3g). Formal correlative analysis of the HD and DMOG treated-monocyte cultures at 10 h post-culture revealed a high degree of correlation amongst the enriched '50' Hallmark gene sets in both treatment types (Spearman’s *ρ* = 0.73, *p* = 9.7e-09, Additional file 3: Fig. S3a). Further analysis of Log2 fold changes revealed that *FCGR2B* gene transcripts were differentially expressed at 10 h post-treatment with DMOG and remained elevated at 24 h post-treatment (Fig. 3h). Immunophenotyping of these monocyte samples using flow cytometry revealed cell surface expression changes in proteins concordant with the RNA-Seq transcriptional data; HLA-DR expression was significantly reduced on DMOG treated monocytes (*p* < 0.01 additional file 3: Fig. S3b). GSEA confirmed Winter Hypoxia Metagene gene set and Hypoxia gene set enrichment at 10 h post-treatment with DMOG (Fig. 3i-j). We further assessed expression of genes which regulate glycolysis, angiogenesis and prolyl hydroxylases, showing these to be upregulated and coincident with increased expression of downstream targets; *P4HA1*, *ENO1*, *GLUT1*, *VEGFA*, *EGLN1* and *EGNL3*. Furthermore, transcription factors *ARNT* and AP-1 encoding genes *JUN* and *FOSL2* were also upregulated (Fig. 3k). *HIF1A* mRNA was itself downregulated (Fig. 3k), a known regulatory response to HIF-1α protein stabilization during hypoxia [81, 82]. The other major HIF; *HIF2A* (*EPAS1*) was not significantly differentially expressed in DMOG treated monocytes (Additional file 3: Fig. S3C). However, IPA was used to identify the top 50 upstream transcription factor regulators of differentially expressed genes, revealing that multiple hypoxia pathway genes were evident, with *HIF1A*, *HIF2A* (*EPAS1*), and *ARNT* (*HIF1B*) present in the top 10. Furthermore, *JUN* and *FOS* which encode proteins that form the AP-1 transcription factor complex, were also identified within the top 50 (Fig. 3i). GSEA revealed a hypoxia gene signature that was detectable at 2 h and sustained through to the 24 h time period post-DMOG treatment (Additional file 3: Fig. S3d). *HIF1A* and *HIF2A* were amongst the top 10 upstream regulators of differentially expressed genes at both the 10- and 24-h time points post-treatment with DMOG (Additional file 3: Fig. S3e).

**Fig. 4:**
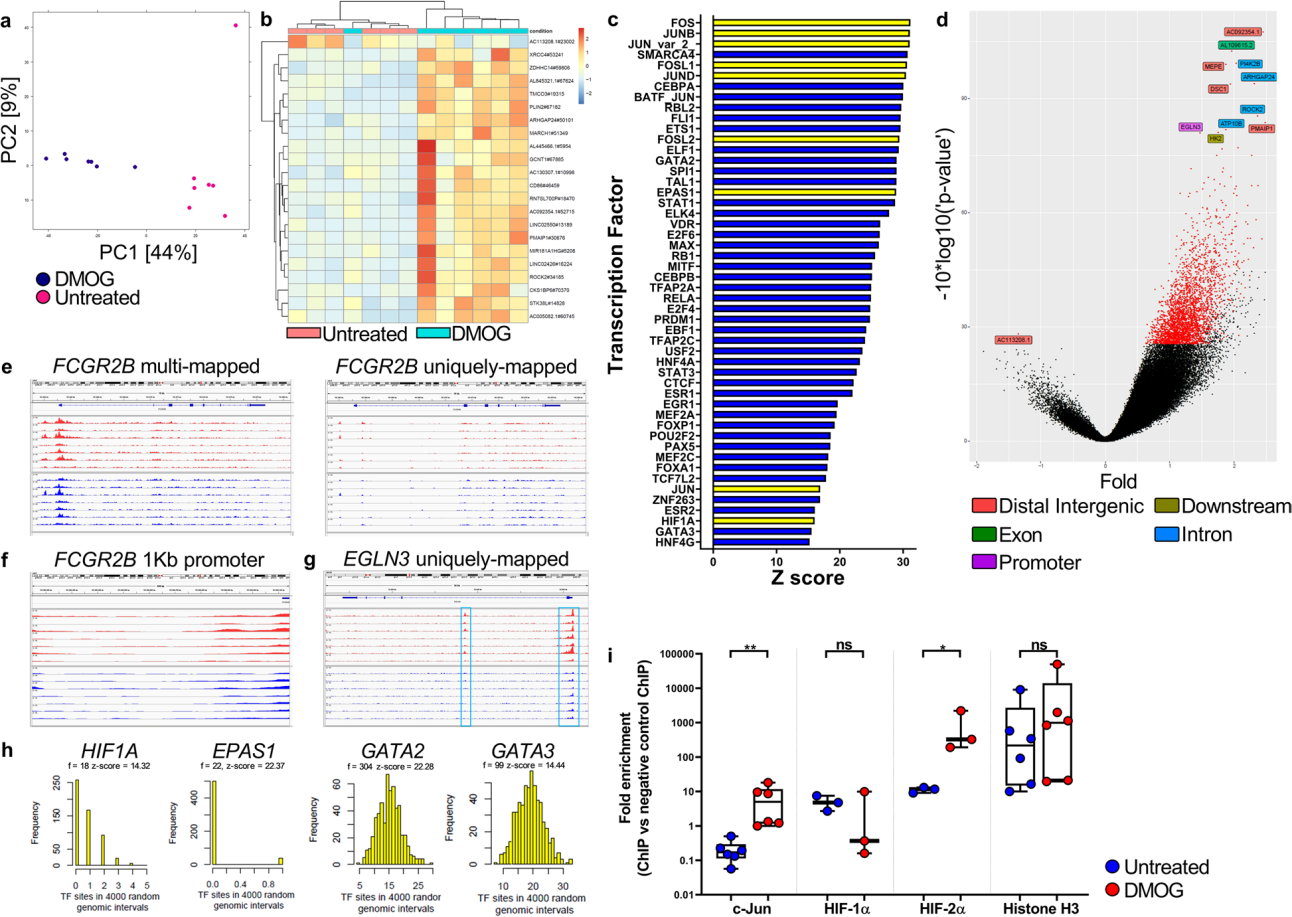
Characterisation of gene openness and transcriptional regulation of *FCGR2B* gene in response to DMOG treatment. **a**, DNA from untreated and DMOG treated monocytes 24 h post-treatment was assessed to determine chromatin accessibility. PCA of gene accessibility in differentially open genes for untreated versus DMOG-treated samples, 24 h post-treatment. **b**, Hierarchical cluster analysis heat map of sample dissimilarity based on significantly opened or closed regions (*n* = 7 per group). **c**, Transcription factor DNA binding motifs identified in significantly opened regions in DMOG treated versus untreated samples. HIFs and AP-1 proteins are colored yellow. **d**, Volcano plot showing genes significantly opened or closed in DMOG-treated monocytes when compared to untreated donor matched monocytes. **e**, Gene coverage tracks of *FCGR2B* at the 10-h time point post-treatment. Gene tracks represent unique donors. DMOG-treated monocyte samples are coloured red and donor matched untreated samples are coloured blue (*n* = 7 per group). **f**, Gene coverage tracks of the 1 Kb region upstream of *FCGR2B* TSS for ATAC-seq alignments, 10 h post-treatment. **g**, Gene coverage track for the *EGLN3* gene, 10 h post-treatment. **h**, Frequency (f) of TF binding sites in differentially open peaks between DMOG treated and untreated monocytes. Z-scores for this observed frequency in relation to the frequency distribution of TF binding occurrences in 4000 random genomic intervals, repeated 1000 times. Frequency distributions for the number of HIF-1α, HIF-2α, GATA2 and GATA3 binding sites are shown. Frequency (f) of TF binding sites. **i**, Box and whisker chart showing ChIP–quantitative PCR confirmation of TF binding to the 1 Kb region upstream of the *FCGR2B* TSS in the promotor region. Plots show all mAb binding and subsequent PCR amplification of the *FCGR2B* gene promotor region normalized to the signal achieved in the donor matched isotype control mAb ‘ChIPed’ DNA samples (*n* = 3–6 per group). Statistical significance was assessed using a paired two-tailed Wilcoxon test (*p < 0.05, **p < 0.01 and ns = non-significant). Also see Additional file 4: Fig. S4

We next profiled chromatin accessibility in untreated and DMOG-treated primary human monocytes using ATAC-seq (using material from the same experiment reported in Fig. 3). Chromatin accessibility analysis of DNA from 24 h post-treatment, revealed considerable separation, when comparing differentially open and closed regions of the genome between untreated and DMOG treated monocytes as viewed by PCA (Fig. 4a). Furthermore, hierarchical clustering analysis of significantly differentially open and closed regions revealed marked dissimilarity between donor matched untreated and DMOG treated monocytes (Fig. 4b). Canonical transcription factor DNA binding motifs were next quantified within significantly opened regions in DMOG treated samples when compared to untreated samples. HIF-1α, HIF-2α and proteins which form the AP-1 transcription factor complex were amongst the top 50 transcription factors predicted to access more open regions of the genome after DMOG treatment (Fig. 4c). Amongst the most significantly open genomic regions in DMOG treated monocytes when compared to donor matched untreated monocytes, was the promotor region of *EGNL3*, which encodes PHD2 and is known to be upregulated in response to HIF-α protein stabilization (Fig. 4d). We also looked for DMOG-induced changes directly in the low affinity *FCGR* locus. *FCGR2B* and *FCGR2C* share sequence homology resulting in considerable multi-mapping for these two genes. Nonetheless, when comparing both multi-mapped and uniquely-mapped reads for *FCGR2B* we observed no significantly open genomic regions (peaks) between untreated and DMOG treated monocytes (Fig. 4e). Furthermore, visualization of the multi-mapped 1 Kb region upstream of the *FCGR2B* gene transcription start site (TSS), did not reveal additional or more pronounced peaks (Fig. 4f). In contrast, additional and pronounced peaks were observed for *EGLN3* (which was one of the most significantly open genes in DMOG treated monocytes; Fig. 4g). This indicated that the enhancement of *FCGR2B* gene expression in DMOG treated monocytes was not mediated by increased chromatin accessibility within the *FCGR2B* gene locus, but instead was likely driven by altered transcription factor binding. Open peaks were scanned for transcription factor binding sites using the ORegAnno database, in differentially regulated regions of the genome when comparing untreated versus DMOG-treated monocytes. This analysis revealed a non-random distribution for HIF-1α and HIF-2α binding motifs, in contrast to GATA-binding factor 2 and GATA-binding factor 3 binding motifs which were randomly distributed in open regions of the genome (Fig. 4h). To determine whether HIFs and AP-1 could directly interact with the *FCGR2B* gene promotor, we first searched for the HIF-1β/HIF-α and AP-1 canonical core motifs (as defined by the JASPAR open-access database for TF binding profiles, Additional file 4: Fig. S4a), in the 1 Kb region upstream of the *FCGR2B* gene TSS, however, precise matches were not located within this region. Olferiev et al., previously described a non-canonical motif for AP-1 in the *FCGR2B* gene promotor [69] and we also located this motif at position -339 upstream of the TSS. Additionally, we also identified a non-canonical hypoxia response element (HRE) and a potential HIF binding motif at position -835 (Additional file 4: Fig. S4b). Similar non-canonical HIF binding motifs have been previously reported for *CD73* [83] and *PEPCK* [84]. To confirm whether these molecules were enriched for binding to the *FCGR2B* gene promotor during DMOG treatment we performed ChIP. Using specific mAb for c-Jun, HIF-1α, and HIF-2α we performed ChIP-quantitative PCR analysis on mAb extracted DNA to detect the *FCGR2B* promotor region (normalized with the negative isotype control mAb) revealing that only c-Jun and HIF-2a increasingly interact directly with the *FCGR2B* gene promotor region at 24 h post-DMOG treatment (Fig. 4i). Additionally, we also analyzed ChIP-Seq data generated by Tausendschön et al., who utilized HIF-1α and HIF-2α specific mAbs, to determine genomic HIF-α interactions in human MDMs cultured at 1% O_2_ for 8 h [60]. At this earlier time point (our ChIP assay was carried out using monocytes cultured for 24 h), a peak within the 10 Kb region upstream of the *FCGR2B* gene was detected in both anti-HIF-1α and HIF-2α ‘ChIPed’ DNA of hypoxic but not normoxic MDMs, but no HIF-α interaction was detected in the 1 Kb region upstream of the *FCGR2B* gene TSS (Additional file 4: Fig. S4c). Tausendschön et al., also knocked down *HIF1A* and *HIF2A* genes in normoxic and hypoxic human MDMs using siRNA [60] and our analysis of this data also revealed downregulation of *FCGR2B* expression, particularly following *HIF2A* knockdown in hypoxic MDMs (Additional file 4: Fig. S4d). Altogether these data show that DMOG treatment of human monocytes potently induces a hypoxia related gene signature alongside prominent transcriptional modulation by HIFs and AP-1, that is coincident with an enhancement of FcγRIIb expression and consequent downregulation of the FcγR A:I ratio.

### Physiological hypoxia and pharmacological HIF activation lead to comparable enhancement of FcγRIIb expression levels

**Fig. 5:**
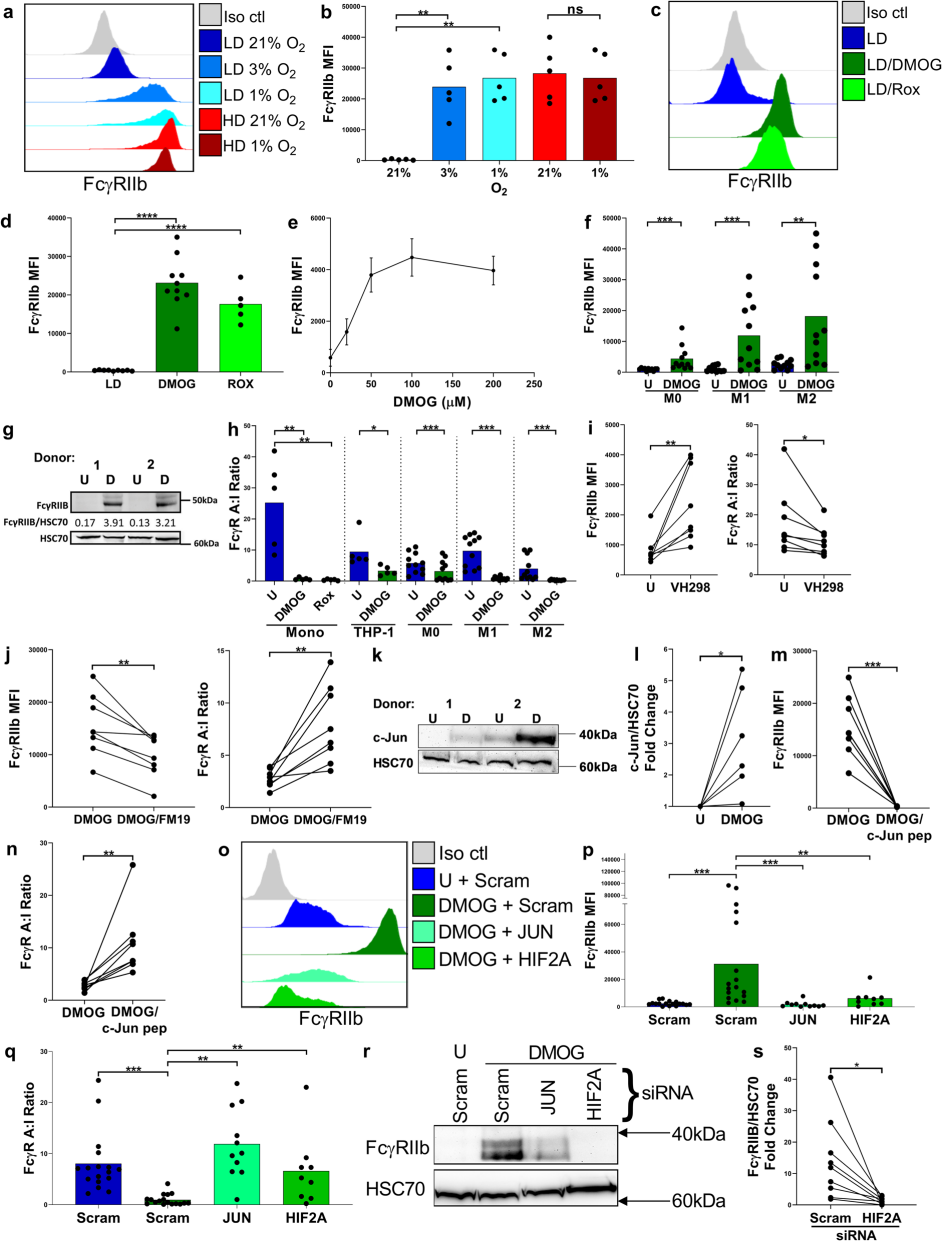
Effects of hypoxia and hypoxia mimetics on FcγRIIb expression and its transcriptional regulation. **a**, Histograms showing expression of FcγRIIb on LD and HD monocytes cultured under 21% or 1% O_2_, **b**, and quantified using flow cytometry (*n* = 5 per group). **c**, Histograms showing expression of FcγRIIb on monocytes treated with DMOG or Roxadustat (Rox). **d**, and quantified using flow cytometry (*n* = 5–10 per group). **e**, FcγRIIb expression quantified using flow cytometry following dose titration of DMOG treatment of THP-1 cells (*n* = 3, bars show means ± SEM). **f**, FcγRIIb expression on untreated (U) and DMOG treated M0, M1 and M2 monocyte-derived macrophages (MDM), (*n* = 11 per group). **g**, Representative Western blot showing FcγRIIb expression in untreated or DMOG-treated (D) M1 macrophages for 2 donors. **h**, FcγR A:I ratio on monocytes, THP-1 cells and MDMs untreated or treated with DMOG or Roxadustat (Rox) (*n* = 5–11 per group). **i**, FcγRIIb expression (left) and FcγR A:I ratio (right) on monocytes following treatment with VH298 (*n* = 8). **j**, FcγRIIb expression (left) and FcγR A:I ratio (right) on DMOG-treated monocytes in the absence or presence of FM19G11 (FM19, *n* = 8). **k**, Representative Western blot showing c-Jun expression in Untreated (U) or DMOG-treated (D) monocytes **l**, combined Western blot data of fold change of c-Jun expression relative to HSC70 (*n* = 6). **m**, FcγRIIb expression and **n**, FcγR A:I ratio on DMOG-treated monocytes following c-Jun peptide treatment (c-Jun; *n* = 8). **o**, Representative histograms showing FcγRIIb expression on purified human untreated (U) monocytes transfected with scrambled control (Scram) siRNA, and DMOG-treated monocytes transfected with Scram, *HIF2A* or *JUN* siRNA, 24 h post-treatment. **p**, FcγRIIb expression and **q**, FcγR A:I ratio for 9–17 donors per group using flow cytometry following treatments stated in **o**. **r**, Representative Western blots showing FcγRIIb expression on untreated monocytes transfected with Scram siRNA, and DMOG-treated monocytes transfected with Scram, *HIF2A* or *JUN* siRNA. **s**, Combined Western blot data of fold change of FcγRIIb expression relative to HSC70 (*n* = 9). Each point on the graphs represents a unique donor and bars represent group means. Statistical significance was assessed using a paired two-tailed Wilcoxon test (*p < 0.05, **p < 0.01, ****p < 0.0001 and ns = non-significant). Also see Additional file 5: Fig. S5

To confirm that hypoxic conditions upregulate FcγRIIb on mononuclear phagocytes, we cultured these cells under physiological hypoxia. Human LD monocytes cultured under hypoxic conditions (1% or 3% O_2_) significantly upregulated FcγRIIb when compared to monocytes cultured under normoxic conditions (21% O_2_), (*p* < 0.01, Fig. 5a-b). Treatment of PBMCs with the pan-HIF-PH inhibitor, DMOG, or the prolyl hydroxylase domain 2 (PHD2) inhibitor, Roxadustat [85], led to comparable enhancement of FcγRIIb expression (*p* < 0.0001 for both inhibitors, Fig. 5c-d). DMOG treatment of monocytic THP-1 cells also enhanced FcγRIIb expression in a dose dependent manner (Fig. 5e). Equivalent experiments with various B cell lines saw no change in FcγRIIb expression (data not shown), again underlining differential regulation in B versus myeloid cells. We next differentiated monocytes into macrophages using M-CSF over 7 days and then stimulated them with LPS/IFN-γ (M1), IL4/IL-13 (M2) or left them untreated (M0) for 2 days in the absence or presence of DMOG. DMOG treatment of all three types of MDMs significantly upregulated FcγRIIb, being particularly evident for M1 and M2 macrophages (*p* < 0.001 and *p* < 0.01 for M1 and M2, respectively, Fig. 5f) and confirmed by Western blot (Fig. 5g). Importantly, DMOG treatment of human monocytes, MDMs and THP-1 cells consistently and significantly decreased the FcγR A:I ratio (Fig. 5h), indicating that HIF activation can profoundly alter FcγR expression on mononuclear phagocytes in a manner that may be detrimental to mAb immunotherapy.

### Upregulation of FcγRIIb in human mononuclear phagocytes is mediated by HIFs and AP-1

To further define the mechanism underlying *FCGR2B* upregulation and ascertain the importance of AP-1 and HIFs in enhancing FcγRIIb cell surface expression on mononuclear phagocytes, we first used a series of small molecule inhibitors. Digoxin has been reported to inhibit HIF-1α translation [86, 87] and we observed significant inhibition of FcγRIIb upregulation (*p* < 0.0001) and changes in FcγR A:I ratio (*p* < 0.05) on HD monocytes following treatment with Digoxin (Additional file 5: Fig. S5a-b), supporting a role for HIFs. Furthermore, treatment of LD monocytes with the VHL inhibitor; VH298 [38], which stabilizes HIF-α subunit protein expression, increased FcγRIIb expression (*p* < 0.01) and decreased the FcγR A:I ratio (*p* < 0.05, Fig. 5i). Simultaneous treatment of DMOG treated-monocytes with the HIF-α inhibitor FM19G11 [39], diminished the increase in FcγRIIb expression (*p* < 0.01) and consequently increased the FcγR A:I ratio (*p* < 0.05, Fig. 5j). Significant upregulation of c-Jun protein in DMOG-treated monocytes was confirmed by Western Blot (Fig. 5k-l). Furthermore, culturing DMOG-treated monocytes with a c-Jun peptide inhibitor, which abrogates JNK/c-Jun interactions [40], also led to a potent inhibition of FcγRIIb upregulation, impairing the reduction in FcγR A:I ratio, (Fig. 5m-n). M1 MDMs treated with the c-Jun peptide inhibitor also experienced a similar impairment of change of FcγR expression levels, in response to DMOG treatment (Additional file 5: Fig. S5c-d). These findings indicated that AP-1 and HIFs were involved in the enhancement of FcγRIIb expression on hypoxic mononuclear phagocytes.

However, to more precisely assess their contribution, we used siRNA-mediated knock-down in untreated and DMOG treated monocytes. We first knocked down *HIF1A* in LD, HD and DMOG treated human monocytes using *HIF1A* specific siRNA, confirming the knock down by measuring HIF-1α expression via Western blot (Additional file 5: Fig. S5e). Although ChIP assessment revealed that HIF-1α did not interact with *FCGR2B* gene promotor at 24 h post-DMOG treatment (Fig. 4i), here *HIF1A* knock-down inhibited FcγRIIb upregulation in both HD (Additional file 5: Fig. S5f) and DMOG treated monocytes (Additional file 5: Fig. S5g), confirmed by measuring FcγRIIb expression using flow cytometry. This indicated a non-redundant role for HIF-1α in enhancing FcγRIIb expression on mononuclear phagocytes under hypoxia-like conditions. Next we knocked down *HIF2A* and *JUN* using siRNA, which almost entirely prevented the upregulation of FcγRIIb in DMOG treated monocytes (Fig. 5o-p) and consequently prevented the reduction in the FcγR A:I ratio (Fig. 5q), when assessed by flow cytometry. *HIF2A* and *JUN* knock downs using these siRNA were confirmed by assessing HIF-2α and c-Jun by Western blot (Additional file 5: Fig. S5h). Prevention of FcγRIIb upregulation in DMOG treated monocytes post-*HIF2A* and *JUN* knockdowns were also confirmed by Western blot (Fig. 5r-s). AP-1 activity can be regulated by post-translational modification, including phosphorylation by the mitogen-activated protein kinase (MAPK) family which comprises of MAPKs, the extracellular signal regulated kinase (ERK), p38 MAPK and c-Jun NH2-terminal kinase (JNK) [88]. Therefore, we determined the phosphorylation status of c-Fos (p–c-Fos) in DMOG treated monocytes and observed that it was elevated relative to untreated monocytes (Additional file 5: Fig. S5i), indicating the association of AP-1 activation with HIF-α protein stabilization. These observations led us to conclude that the enhancement of FcγRIIb expression following HIF activation in mononuclear phagocytes is dependent upon the protein expression and activation of HIF-1α, HIF-2α and AP-1, all of which potentially directly interact with the *FCGR2B* gene loci upstream of its TSS.

### Tumor-associated human and murine mononuclear phagocytes are FcγRIIb^bright^

**Fig. 6:**
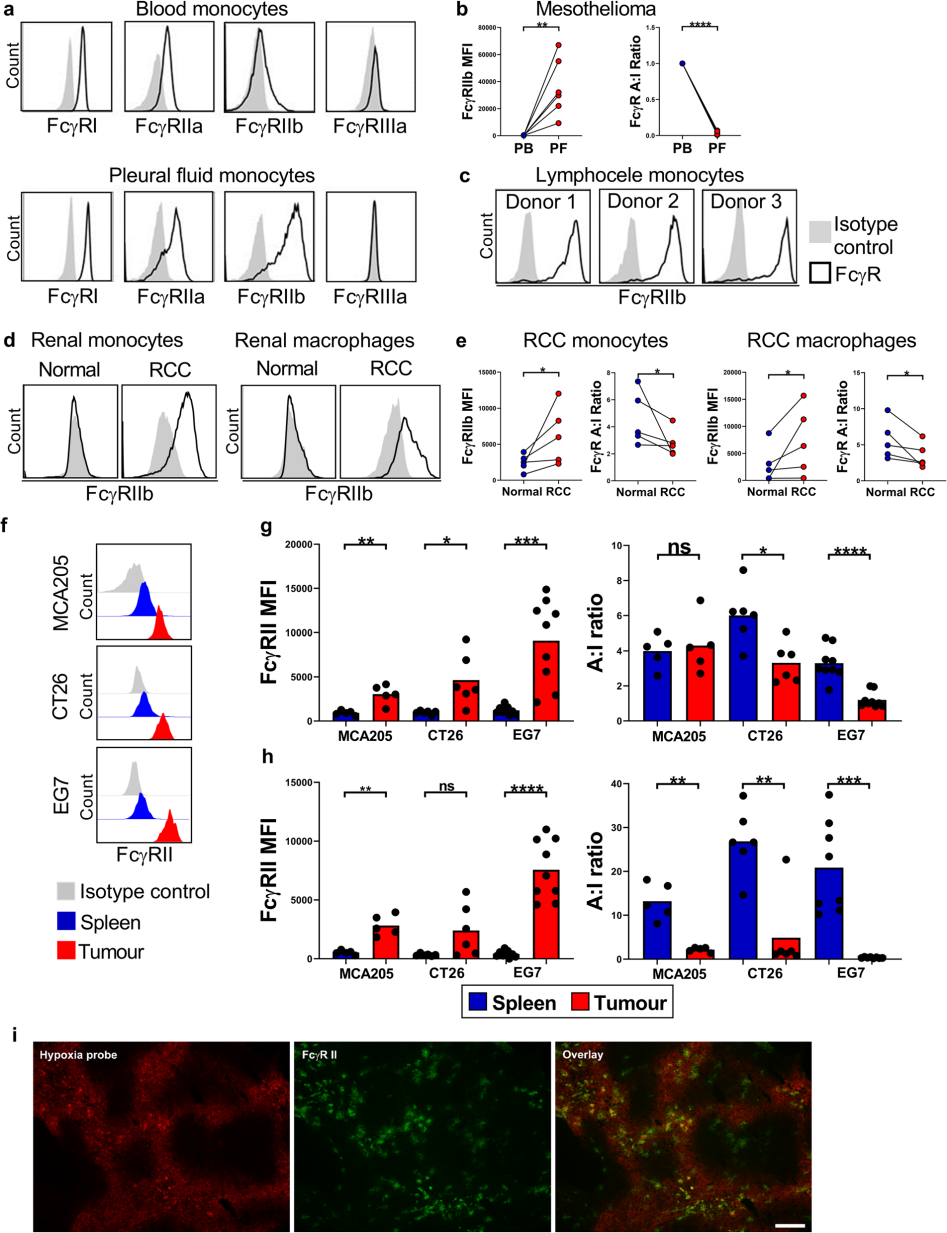
FcγR expression on tumor associated mononuclear phagocytes. **a**, Representative histograms showing FcγR expression on fresh donor matched peripheral blood (PB) and pleural fluid (PF) monocytes from a single mesothelioma patient. **b**, FcγRIIb expression (left) and FcγR A:I ratio (right) were quantified for PB and PF monocytes (FSC^hi^CD45^+^CD14^+^ cells) sourced from mesothelioma patients using flow cytometry (*n* = 6 per group). **c**, Representative histograms showing expression of FcγRIIb on fresh monocytes isolated from lymphocele taken from 3 breast cancer patients. **d**, Representative histograms showing FcγRIIb expression on fresh donor matched renal monocytes (FSChiCD45+CD14+ cells) and macrophages (FSChiCD45+CD163+ cells) in normal kidney tissue and tumor from a single renal cell carcinoma (RCC) patient. **e**, FcγRIIb expression (left) and FcγR A:I ratio (right) were quantified for monocytes and macrophages sourced from normal kidney tissue and donor matched RCC specimens using flow cytometry (*n* = 5 per group). **f**, Representative histograms showing FcγRIIb expression on splenic and MCA205, CT26 and EG7 tumor associated CD11b+F4/80+ macrophages. **g**, Comparison of murine FcγRII expression (left) and FcγR A:I ratio (right) on CD11b+Ly6C+ monocytes and **h**, CD11blo/F4/80+ macrophages in recipient matched spleen and subcutaneous MCA205, CT26 and EG7 tumors (*n* = 5–9 per group). Each point on the graphs represents a unique human subject or mouse and bars represent group means. Statistical significance between groups was assessed using a paired two-tailed Wilcoxon test (*p < 0.05, **p < 0.01, ***p < 0.001, ****p < 0.0001 and ns = non-significant). **i**, Immunofluorescence staining of hypoxic regions using hypoxyprobe (hypoxia probe) and anti-mouse FcγRII, on sections taken from a CT26 tumor. Localization of FcγRII expression in hypoxic regions is shown. Images representative of stained sections from 5 different mice. Scale bars 100 μm. Also see Additional file 6: Fig. S6

To explore the broader relevance of our observations regarding the hypoxia-induced upregulation of FcγRIIb expression on mononuclear phagocytes, we immunophenotyped these cell types in contexts where hypoxia is likely present such as within human and murine tumors, and tumor associated ascites. First, we compared FcγRIIb expression on peripheral blood and pleural fluid monocytes from mesothelioma patients where the oxygen levels would be expected to differ [89]. Monocytes in the pleural fluid of these patients expressed significantly elevated levels of FcγRIIb relative to donor matched peripheral blood monocytes (*p* < 0.01, Fig. 6a-b). Pleural fluid monocytes also possessed a significantly lower FcγR A:I ratio in comparison to peripheral blood monocytes (*p* < 0.0001, Fig. 6b). Pleural fluid and peripheral blood neutrophils were negative for FcγRIIb and B cells expressed similar levels of FcγRIIb in both niches (data not shown). FcγRIIb^bright^ monocytes were also detected in the ascites of breast cancer patients (Fig. 6c). In renal cell carcinoma (RCC), a tumor type associated with high HIF-α expression [90], both monocytes and macrophages expressed significantly elevated levels of FcγRIIb, relative to donor matched counterparts in healthy kidney tissue (*p* < 0.05, Fig. 6d-e). When comparing splenic and tumor FcγRII expression in WT C57BL/6 J mice, expression was elevated in all three subcutaneous tumor models we examined (MCA205, CT26 and EG7). FcγRII expression was significantly elevated on CD11b^+^Ly6C^hi^ monocytes, in mice inoculated with the MCA205 (*p* < 0.01), CT26 (*p* < 0.05) and EG7 subcutaneous tumors (*p* < 0.0001), consequently reducing FcγR A:I ratios on monocytes in CT26 (*p* < 0.05) and EG7 (*p* < 0.0001) tumors relative to matched spleens (Fig. 6g). Tumor associated F4/80^+^ macrophages in mice inoculated with the MCA205 (*p* < 0.01), CT26 (*p* = 0.062) and EG7 (*p* < 0.0001) also expressed elevated levels of FcγRII when compared to matched splenic F4/80^+^ cells, with the FcγR A:I ratio similarly and significantly reduced in MCA205 (*p* < 0.01), CT26 (*p* < 0.01) and EG7 (*p* < 0.001) tumors (Fig. 6h). To investigate the expression of FcγRII in hypoxic regions of tumors we utilized immunofluorescence microscopy. Hypoxic regions of tumor sections were identified using Hypoxyprobe (pimonidazole) and co-localization with FcγRII was assessed. These studies revealed FcγRII expression was concurrent with hypoxic regions of the tumor within three different tumor models: CT26 tumors (Fig. 6i), MC38 and EO771 (Additional file 6: Fig. S6a-b). These observations indicate that FcγRIIb expression on mononuclear phagocytes is elevated when they are associated with, or resident within, human and murine tumors, where it profoundly impacts the FcγR A:I ratio. We hypothesized that the FcγRIIb^bright^ phenotype of these tumor associated mononuclear phagocytes had the potential to impair direct targeting mAb immunotherapy.

### mAb-mediated phagocytic function is impaired in FcγRIIb^bright^ mononuclear phagocytes

**Fig. 7:**
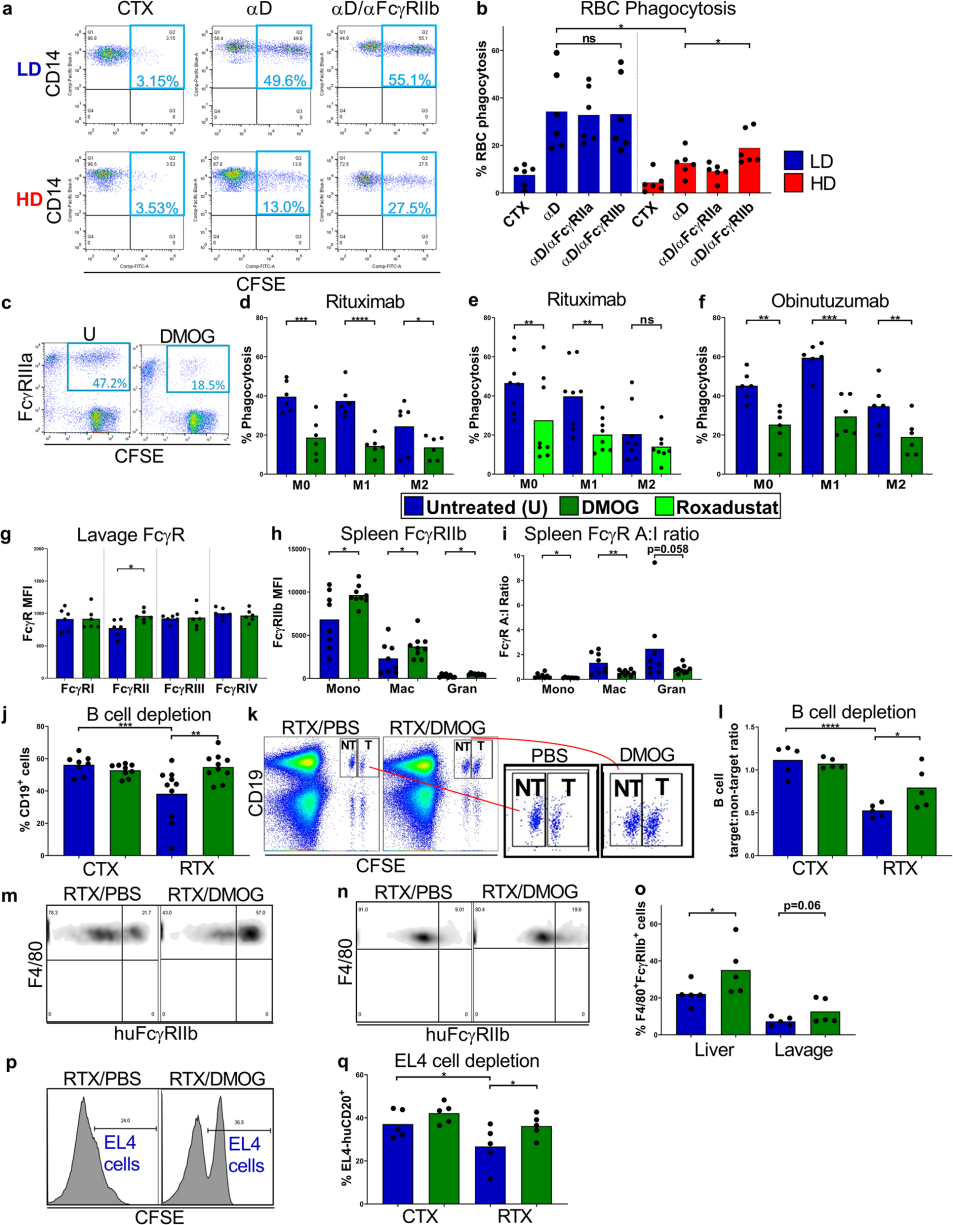
The impact of hypoxia-driven FcγRIIb upregulation on mAb mediated target cell depletion. **a**, Flow cytometry plots showing levels of uptake of CSFE^+^ red blood cells (RBCs) by LD and HD monocytes. RBCs sourced from Rhesus D^+^ individuals were opsonised with control cetuximab (CTX) or anti-Rhesus D antigen specific mAb (αD). RBCs used as targets for LD and HD pre-cultured monocytes pre-treated with or without anti-FcγRIIb (αFcγRIIb) blocking mAb. **b**, RBC phagocytosis quantified for 6 donors. **c**, Flow cytometry plots showing Rituximab mediated uptake of CLL cells by FcγRIIIa^+^ M1 macrophages generated with or without DMOG. **d**, CLL cells opsonised with Rituximab and cultured with M0, M1 or M2 MDMs generated in the absence or presence of DMOG or **e**, Roxadustat and the percentage of phagocytic MDMs were determined by flow cytometry (*n* = 6–8 per group). **f**, Phagocytosis of CLL cells mediated by Obinutuzumab (*n* = 6 per group). **g**, FcγR expression on F4/80^+^ macrophages in the peritoneal lavage of WT C57BL/6 mice treated with DMOG or PBS control. i.p., determined using flow cytometry (*n* = 6 per group). **h**, FcγRIIb expression levels and **i**, FcγR A:I ratio were determined by flow cytometry in splenic monocytes (Mono), macrophages (Mac) and granulocytes (Gran) of DMOG or PBS treated hFcγRIIb/mFcγRIIKO mice (*n* = 8 per group). **j**, hFcγRIIb/mFcγRIIKO/hCD20 mice were treated with DMOG or vehicle PBS control i.p. for 72 h prior to receiving Rituximab (RTX) or CTX isotype control. %CD19^+^ cells in the peripheral blood of each mouse were determined using flow cytometry (*n* = 8–10 per group). **k**, hFcγRIIb/mFcγRIIKO mice were treated with DMOG or PBS i.p. for 72 h prior to receiving CFSE labelled target splenocytes from hCD20/mFcγRIIKO mice and non-target splenocytes from WT C57BL/6 mice, i.v. These mice were treated with DMOG or PBS i.p. prior to receiving RTX or CTX 24 h later. Flow cytometry plots are shown for the depletion of target and non-target splenocytes, and **l**, data is presented as CD19^+^ cell target:non-target ratio (*n* = 5 per group). **m**, Flow cytometry plots showing hFcγRIIb expression on liver and **n**, peritoneal lavage F4/80^+^ macrophages 72 h post-treatment with DMOG or PBS control, i.p., in hFcγRIIb/mFcγRIIKO mice, and **o**, quantified for 5 mice per group. **p**, hFcγRIIb/mFcγRIIKO mice were treated with DMOG or PBS i.p. for 60 h prior to receiving CFSE labelled EL4-huCD20^+^ cells following treatment with RTX or CTX, and DMOG or PBS. Histograms showing depletion of target EL4-huCD20^+^ cells in the peritoneum by RTX in the absence and presence of DMOG and **q**, EL4-huCD20^+^ cell depletion in the peritoneal lavage quantified using flow cytometry. Bars represent group means. Statistical significance was assessed using an unpaired two-tailed t-test, a paired two-tailed Wilcoxon test, or a one-way ANOVA for the in vivo cell depletion experiments (*p < 0.05, **p < 0.01, ***p < 0.001, ****p < 0.0001 and ns = non- significant). Also see Additional file 7: Fig. S7

We next sought to determine the functional consequence of FcγRIIb upregulation on mononuclear phagocytes. To investigate this, we assessed the ability of HD versus LD human monocytes to phagocytose RBCs opsonized with anti-D mAb. We observed that the phagocytic function of HD monocytes was significantly diminished in comparison to LD monocytes (*p* < 0.05, Fig. 7a and b). When FcγRIIb on HD monocytes was blocked using a F(ab’)_2_ FcγRIIb specific antibody, phagocytic function was significantly improved (*p* < 0.05) unlike when the activating FcγRIIa was blocked (Fig. 7a-b).

Next, we examined the impact of hypoxia on MDMs and used untreated and HIF-PHD inhibitor-treated M0, M1 and M2 MDMs as effector cells in ADCP assays. We observed phagocytosis of Rituximab opsonized CLL cells was significantly decreased by DMOG or roxadustat treatment in M0, M1 and M2 MDMs (Fig. 7c-e). This significant reduction in ADCP function of DMOG-treated MDMs was also observed when CLL cells were opsonized with another anti-CD20 mAb, obinutuzumab (Fig. 7f).

Having established these significant effects in vitro, we next explored the effects of hypoxia induction, using HIF-PH inhibition, on FcγR expression and target cell depletion in vivo. In wild type C57BL/6 J mice, DMOG introduction into the peritoneum significantly increased FcγRII expression on macrophages (Fig. 7g) and monocytes (Additional file 7: Fig. S7a-b). Similar effects were seen with Roxadustat (Additional file 7: Fig. S7c-d). Furthermore, HIF-PH inhibitor treatment also enhanced FcγRII expression on peripheral blood monocytes and on splenic monocytes and macrophages in wild type C57BL/6 J mice (Additional file 7: Fig. S7e-g).

Having established the ability of these HIF-PH inhibitors to mediate in vivo changes in FcγR expression and A:I ratio, we assessed the impact of these changes on mAb-mediated target cell deletion. Accordingly, Roxadustat was administered to C57BL/6 J mice before treatment with the potent anti-mCD20 mAb; 18B12 [36] and B cell deletion was assessed. Roxadustat evoked a significant impairment in B cell deletion (Additional file 7: Fig. S7h). Furthermore, Rituximab mediated depletion of human CD20^+^ (hCD20^+^) EL4 tumor cells in the peritoneum of roxadustat treated C57BL/6 J mice was also significantly impaired (*p* < 0.05, additional file 7: Fig. S7i-j). To extend the translational relevance of our findings, we next assessed the effects of HIF-PH inhibition on human FcγRIIb (hFcγRIIb) expression and mAb mediated target depletion in transgenic mice expressing the human *FCGR2B* and *CD20* genes and lacking the murine FcγRII (hFcγRIIb^+/−^ x mFcγRII^−/−^ x hCD20^+/−^mice). DMOG treatment in these mice resulted in significant increases of hFcγRIIb expression on monocytes, macrophages and neutrophils in the spleen (Fig. 7h). Significant decreases in the FcγR A:I ratio, because of DMOG mediated enhancement of FcγRIIb expression, were also observed in splenic monocytes and macrophages (Fig. 7i). Rituximab-mediated splenic B cell depletion was significantly impaired in DMOG-treated transgenic hFcγRIIb^+/−^ x mFcγRII^−/−^ x hCD20^+/−^ mice (Fig. 7j). To refine this in vivo model, we adoptively transferred wild type (non-target) or transgenic hCD20^+^ splenocytes (target) into hFcγRIIb^+/−^ x mFcγRII^−/−^ mice. We observed rituximab-mediated depletion of the huCD20^+^ B cells was impaired post-DMOG treatment, whereas the non-target wild type B cell frequencies remained constant across all treatment groups (*p* < 0.05, Fig. 7k-l). Moreover, hFcγRIIb expression on liver macrophages was significantly elevated in DMOG treated hFcγRIIb^+/−^ x mFcγRII^−/−^ mice (*p* < 0.05) with a similar trend for peritoneal macrophages (*p* = 0.06, Fig. 7m-o). Finally, we assessed rituximab mediated depletion of malignant hCD20^+^ EL4 tumor cells from the peritoneum of hFcγRIIb^+/−^ x mFcγRII^−/−^ mice. We observed that DMOG treatment also significantly impaired target cell depletion in this model (*p* < 0.05, Fig. 7p-q). In summary, HIF activation via HD culture or HIF-PHD inhibition significantly impairs the ability of monocytes and macrophages to phagocytose and deplete mAb-opsonized cellular targets in vitro and diminishes direct targeting anti-cancer mAb therapy in vivo.

## Discussion

We demonstrate that exposure to physiological or pharmacological hypoxia induces rapid upregulation of the inhibitory IgG Fc receptor, FcγRIIb, on mononuclear phagocytes. This enhancement of FcγRIIb expression, diminishes the FcγR A:I ratio, consequently impairing the ability of monocytes and macrophages to phagocytose mAb opsonized cancer cells and cellular targets. The generation of these ‘FcγRIIb^bright^’ mononuclear phagocytes under hypoxic conditions is transcriptionally driven and is dependent upon AP-1, as well as HIF-1α and HIF-2α interactions with the *FCGR2B* gene promotor region. Detection of FcγRIIb^bright^ mononuclear phagocytes resident within tumors or in associated niches asserts that these cells may be crucial determinants in reducing the efficacy of widely used direct-targeting mAbs. Our findings highlight a novel mononuclear phagocyte phenotype that in addition to being fostered by the hypoxic TME may be actively selected in rapidly growing solid malignancies thereby diminishing the efficacy of mAb immunotherapies.

We observed that under HD conditions or HIF-PH inhibition, human monocytes rapidly upregulate FcγRIIb, acquiring an FcγRIIb^bright^ phenotype, to display levels exceeding other abundantly expressed surface antigens such as MHC Class I. Furthermore, monocytes obtained from RCC patients or tumor associated niches, such as in the pleural cavity of mesothelioma patients or breast cancer patient ascites, also possess an FcγRIIb^bright^ phenotype, contending that this phenotype is physiologically relevant. In vitro, we primarily modelled the effects of hypoxia on human monocytes, using HD cell culture (in which O_2_ levels rapidly drop to as low as 0.1%) and treatment with the HIF-α protein stabilising reagent; DMOG. It has previously been shown that there is a high degree of concordance between HIF-α binding in human proximal tubular epithelial HKC-8 cells exposed to DMOG and those cultured at 1% O_2,_ where both stimuli produce comparable genome-wide patterns of HIF DNA-binding [91]. GSEA of HD and DMOG treated monocyte transcriptomes also revealed excellent concordance with hypoxia gene signatures, that were amongst the most prominent and coincident with the upregulation of *FCGR2B* expression.

Similar hypoxia-correlated FcγRIIb upregulation was also seen in macrophages and TAMs, which holds further translational significance, as these cells are the key effector mononuclear phagocyte populations with respect to therapeutic mAb-mediated elimination of cancer cells [22, 93, 94]. Macrophages abundantly infiltrate tumors and are found in normoxic and hypoxic tumor compartments, albeit in different polarization states [95]. We observed elevated FcγRIIb expression on macrophages in human RCC and 4 different syngeneic murine subcutaneous tumors spanning colorectal, fibrosarcoma, thymoma and breast cancer models, relative to matched splenic macrophage FcγRIIb expression. Furthermore, HIF-PH inhibitor treatment of WT or hFcγRIIb Tg mice upregulated FcγRIIb on mononuclear phagocytes in vivo. We propose that this FcγRIIb^bright^ phenotype may represent a key determinant of resistance to mAb therapy in the TME. However, hypoxia alone is unlikely to be the only stimulus influencing macrophage behaviour within the TME [96], and the integrated effects of hypoxia, cytokines and multiple other interactions will ultimately shape macrophage phenotype and function. Indeed, hypoxia is a common feature in many pathophysiological states [97–99] in which the respective macrophage phenotype might differ. For instance, whereas hypoxic TAMs are more immunosuppressive, TLR-signalling in sepsis might be expected to induce strong cellular activation even in the presence of hypoxia [17, 100]. Nonetheless, at least with MDMs we observed that DMOG treatment upregulated FcγRIIb on all three types of macrophage polarisation states (M0, M1 and M2) we examined, perhaps indicating that hypoxia may have a powerful and pervasive diminishing effect on FcγR A:I ratio and therefore ADCP.

*HIF1A*, *HIF2A* and *JUN* gene knockdowns revealed that both HIF-α subunits and c-Jun have roles in mediating hypoxia-mediated *FCGR2B* gene expression on mononuclear phagocytes. This data is supported by observations in HeLa cells following exposure to hypoxia [101] where AP-1 transcriptional activity is increased, and AP-1 and HIF-1α binding is required in close proximity for the induction of up to ~ 20% of the HIF binding sites in hypoxic human MDMs [60]. Olferiev et al., have previously reported that AP-1 family members bind to the *FCGR2B* promoter in PMA/ionomycin activated CL-01 and U937 cells [69]. Using ChIP assays followed by PCR amplification, we also observed that c-Jun interacted with the *FCGR2B* gene promotor region containing the non-canonical AP-1 motif; TGCATCA (at -345 upstream of the TSS), in DMOG-treated monocytes. The interaction of AP-1 transcription factors with another non-canonical motif; TGCGTCA contained in the *HLA-DR* gene promoter in a B-cell lymphoma line, provides a further example that AP-1 is capable of interacting with non-canonical consensus DNA sequences and inducing gene expression [102]. We also observed that DMOG-treated monocytes express higher levels of c-Jun protein and RNA-Seq analysis revealed that expression of AP-1 components, *JUN* and *FOS,* also increases post-DMOG treatment.

We further investigated whether HIFs themselves induce *FCGR2B* transcription. In MCF-7 human breast cancer cells, both HIF-1α and HIF-2α primarily bind relatively GC rich DNAse1 sensitive genomic regions, reflecting the concentration of hypoxia response elements (HRE) within chromatin accessible promoter regions and over 500 such HIF-binding sites have been identified across the human genome [92, 103]. HIFs primarily mediate gene expression by binding to HREs, a gene sequence which contains the RCGTG core motif (with preference of A over G at the R position), beyond which a preference is also observed for a CAC motif [91]. We sought to determine whether the *FCGR2B* promotor region contains HREs and three ACGTC and six GCGTC motifs within the 15 Kb region upstream of the *FCGR2B* TSS were identified (data not shown). Furthermore, our analysis, of publicly available ChIP-seq data, sequencing HIF-1α and HIF-2α bound DNA from normoxic and hypoxic human MDMs [60], revealed HIF-α interaction at distal regions > 10 Kb upstream of the TSS at 8 h post-hypoxia (Additional file 4: Fig. S4c). However, we identified that the nearest canonical HRE (GCGTG) motif to the *FCGR2B* TSS is at position -3916 upstream of the TSS. Moreover, using ChIP assays we also identified a sequence close to the AP-1 binding site (at position -838 upstream of the TSS) to be a potential non-canonical HRE with which HIF-2α (but not HIF-1α) may interact in DMOG-treated monocytes. This motif is a non-canonical CCGTG sequence, which has been previously described for *CD73* [83] and *PEPCK* [84] and additionally a CAC motif is also located in close proximity to this motif (Additional file 4: Fig. S4b). Although a role for HIF-2α in the regulation of *FCGR2B* expression was ascertained by ChIP, *HIF1A* gene knock down also revealed its non-redundant role. It has previously been reported that in murine embryonic fibroblasts initial exposure to hypoxia stimulates expression of c-Jun and transient activation of protein kinase and phosphatase activities that regulate c-Jun/AP-1 activity dependent upon HIF1-α [104]. Evidence for direct cooperation between AP-1 and HIF-1α has been reported for *VEGF* and *TH* which contain functional AP-1 and HRE sites [105, 106]. Here we propose a mechanism by which AP-1, HIF-1α/HIF-1β and HIF-2α/HIF-1β transcription factor complexes cooperate to mediate marked cell surface FcγRIIB upregulation under hypoxic conditions on human mononuclear phagocytes.

Previous studies have shown that monocytes and macrophages are key mediators of cancer cell depletion in therapeutic settings utilising direct-targeting mAbs such as Rituximab, Cetuximab and Herceptin [107–110]. Uchida et al., have demonstrated that anti-CD20 mAb mediated depletion of circulating B cells in mice was dependent upon activating FcγR since B cell depletion was almost entirely lost in FcR common γ-chain-null mice (that lack activating FcγRI, FcγRIII and FcγRIV) and monocytes were identified as the key effector population in this context [20], which we and others confirmed in later studies [21]. In the current study we report that Rituximab meditated ADCP of CLL cells by human MDMs is compromised by HIF-PH inhibitor treatment and that the same treatment compromises anti-CD20 mAb mediated depletion of cell targets in multiple niches in vivo. We attribute these outcomes to the potency of hypoxia-mediated upregulation of mononuclear phagocyte FcγRIIb.

Although inhibitory for direct targeting mAb as indicated, it is likely that these hypoxia-mediated changes in FcγR are not detrimental in all scenarios. For example, FcγRIIb is known to act as a positive regulator of several agonistic mAbs targeting immune receptors such as CD40, OX40, 4-1BB and CD28 by providing higher levels of receptor cross-linking [32, 111–113]. Therefore, hypoxia-mediated upregulation of FcγRIIb in the TME may even serve as an important component of efficacy for these mAb.

Further studies are needed to determine if hypoxia could serve as a prognostic marker for response to different mAb therapies (negatively regulating direct targeting modalities but augmenting agonistic immunomodulatory mAb). Similarly, whether hypoxia can be appropriately modulated to improve such therapies remains to be demonstrated. In clinical settings the lack of an accurate and approved method to evaluate tumor hypoxia accounts for the limited capacity to intervene with a personalized hypoxia-based therapy. HIF-1α is a well-appreciated target for cancer therapies, and drugs that indirectly inhibit hypoxia/HIF-1α signalling such as digoxin and acriflavine, have been reported to have relevant impacts – for example decreasing lung metastasis in an orthotopic breast cancer model [114]. However, efforts to develop highly specific and efficacious small molecule HIF-1α inhibitors have been largely unsuccessful [115]. Nevertheless, alternative methods to modify hypoxic regions within tumors include supplemental oxygen, anti-VEGF therapy and use of the chemotherapeutic reagent, and hypoxia activated prodrug; evofosfamide [116–119], which could all be explored in the context of mAb therapy. Similarly, mAb-mediated blockade of the hypoxia upregulated FcγRIIb on TAMs is an exciting and emerging strategy [30, 120, 121], with demonstration of combination effects with several direct targeting mAb in preclinical models and encouraging recent evidence in the clinic (Jerkeman et al., 2020, article accepted and in press, https://doi.org/10.1182/blood-2020-140219).

It will be important to understand what degree of the tumor hypoxia effects on myeloid (and other) cells can be overcome by blockade of Fc:FcγRIIb interactions, potentially leading to additional TME O_2_ modifying approaches as indicated above. Targeting the tumor myeloid landscape and specifically the FcγRIIb^bright^ phenotype in combination with established direct-targeting mAbs provides a potentially powerful novel strategy to overcome disease resistance to current and evolving antibody immunotherapies.

## Availability of supporting data

Microarray and sequencing data generated in this study are deposited in the Gene Expression Omnibus under the following accession numbers: GSE165643 (Microarray for HD monocytes and B-cells), GSE166100 (ATAC-seq for LD-HD monocytes) and GSE165999 (RNA-seq (GSE165998) and ATAC-seq (GSE165997) for DMOG time course monocytes).

## Supplementary Information

**Additional file 1: **
