# Supplementary material for: HIF activation enhances FcγRIIb expression on mononuclear phagocytes impeding tumor targeting antibody immunotherapy
Source: J Exp Clin Cancer Res. 2022 Apr 7;41:131. doi: 10.1186/s13046-022-02294-5 (PMC8988350; doi:10.1186/s13046-022-02294-5)

Additional file 1: Table S1.

Targeted region	Forward sequence	Reverse sequence
FCGR2B promotor; AP-1 binding site	5'-ATGCTCAATTTCAAGAAGCATCCA-3'	5'-TGAGAAAGGGTGATGCAGGA-3'
FCGR2B promotor; HIF-2α binding site	5'-AGGGAAGGTCCTCACAAGAAT-3'	5'-AGGTTTCGGGTTGAATGCCAG-3'

Fig. S1.

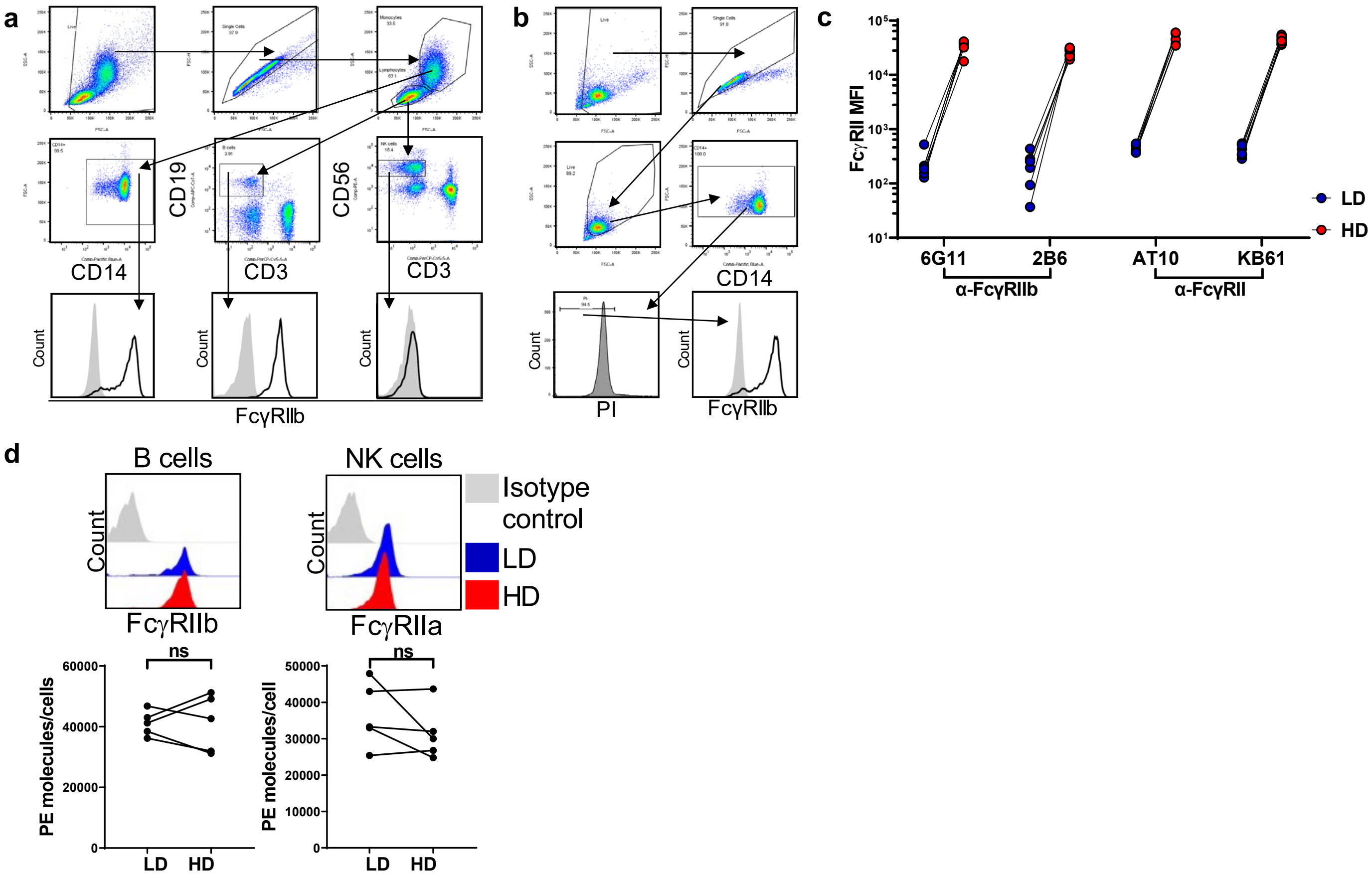

Additional file 2: Fig. S2.

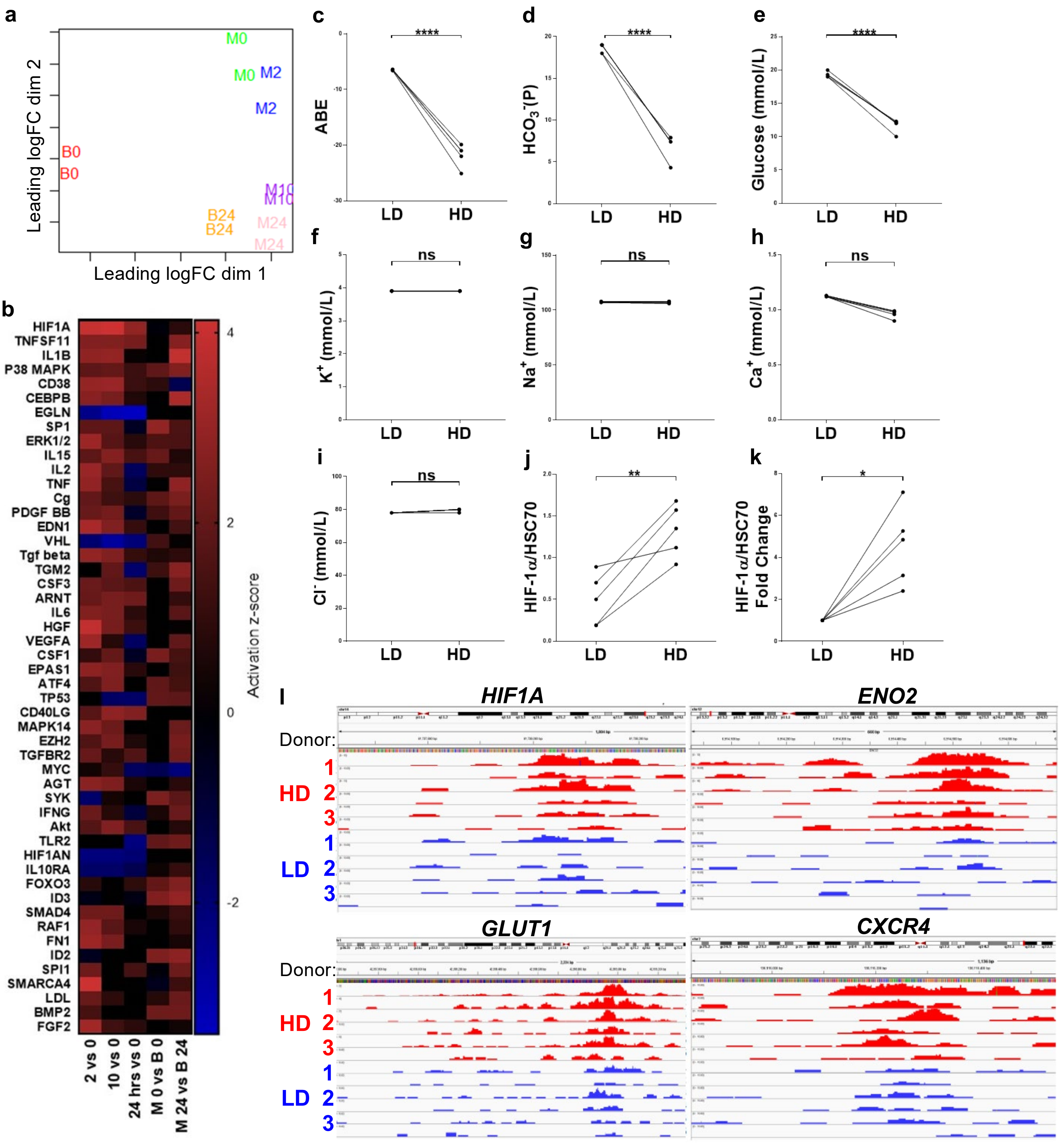

Additional file 3: Fig. S3.

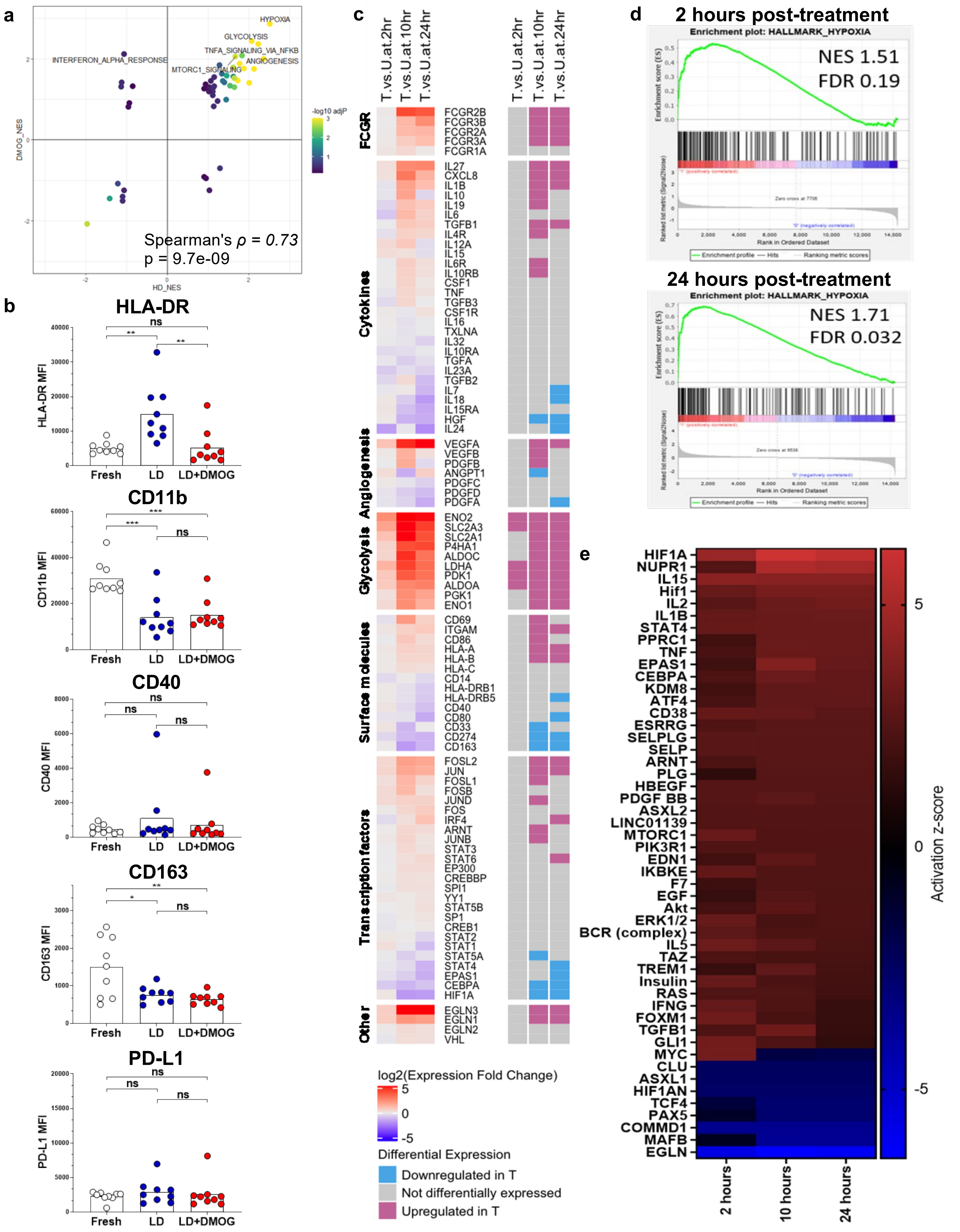

Additional file 4: Fig. S4.

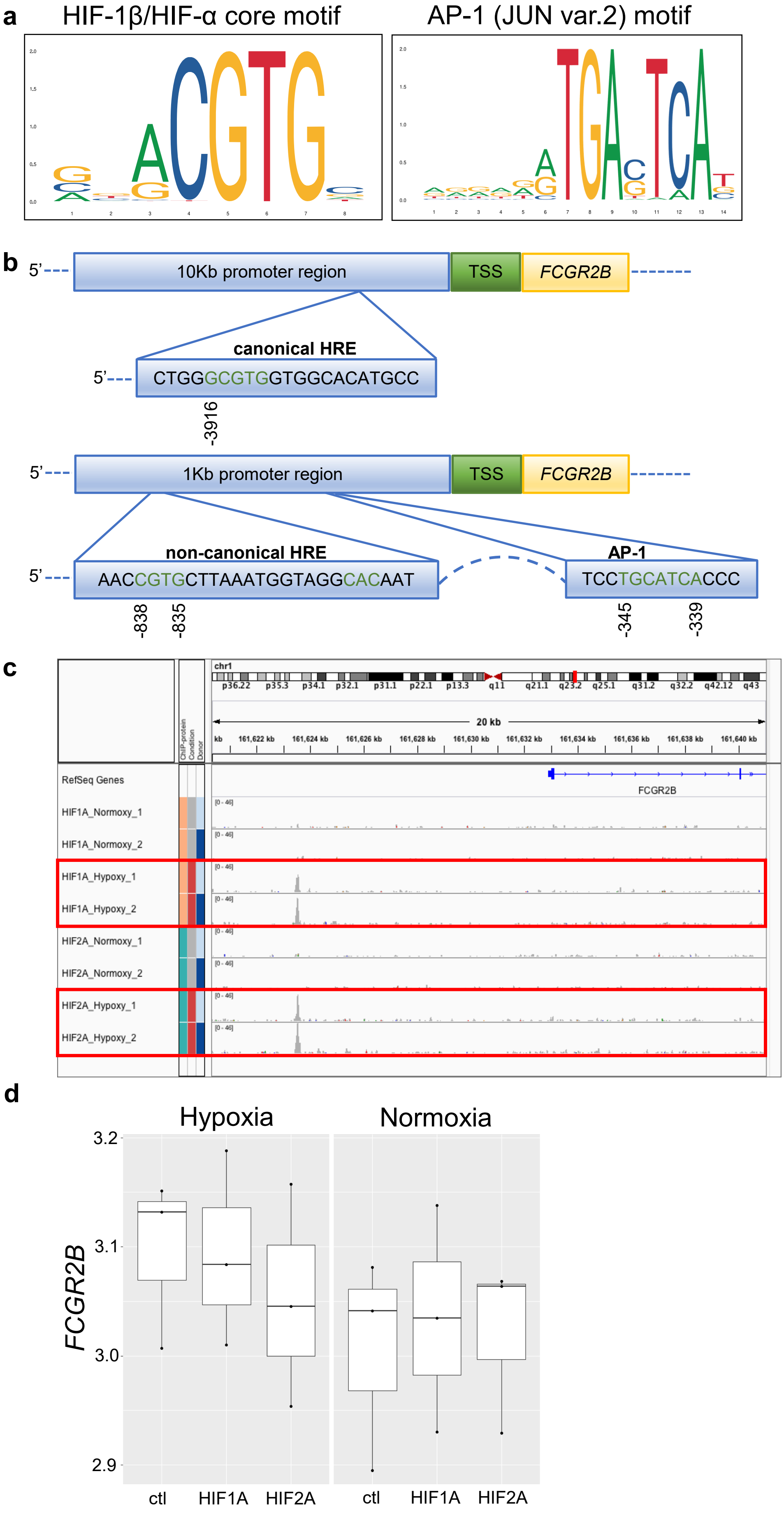

Additional file 5: Fig. S5.

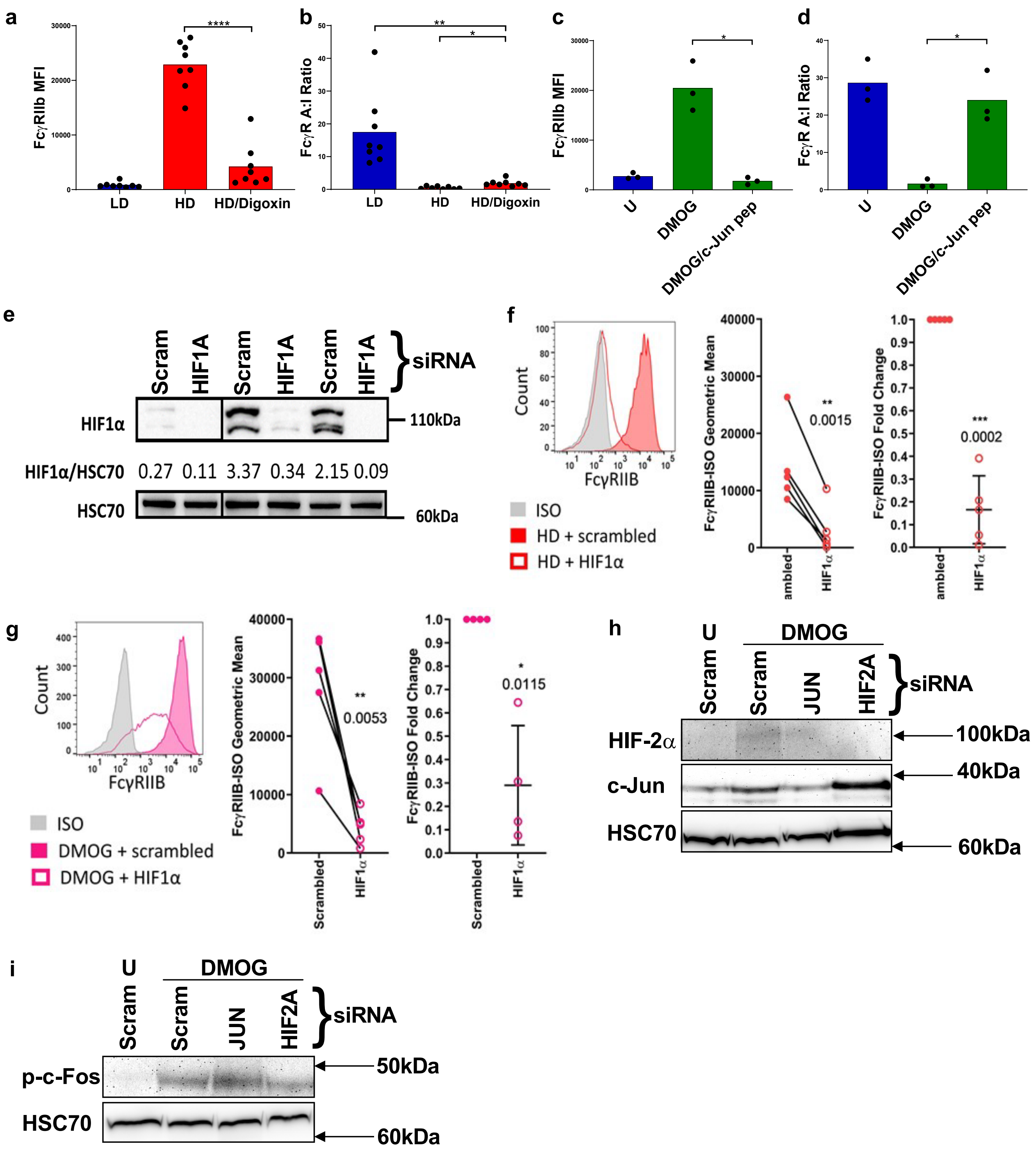

Additional file 6: Fig. S6.

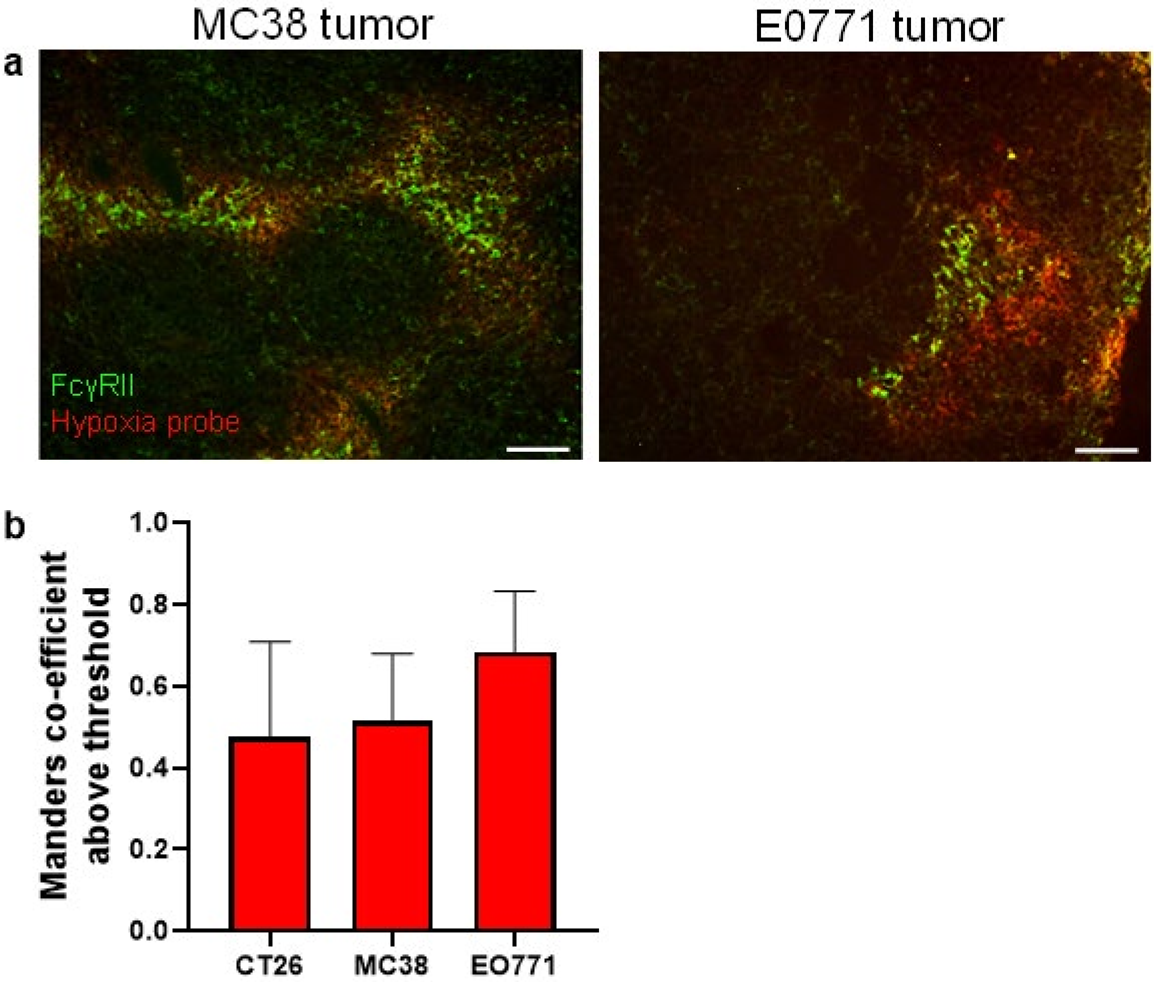

Additional file 7: Fig. S7.

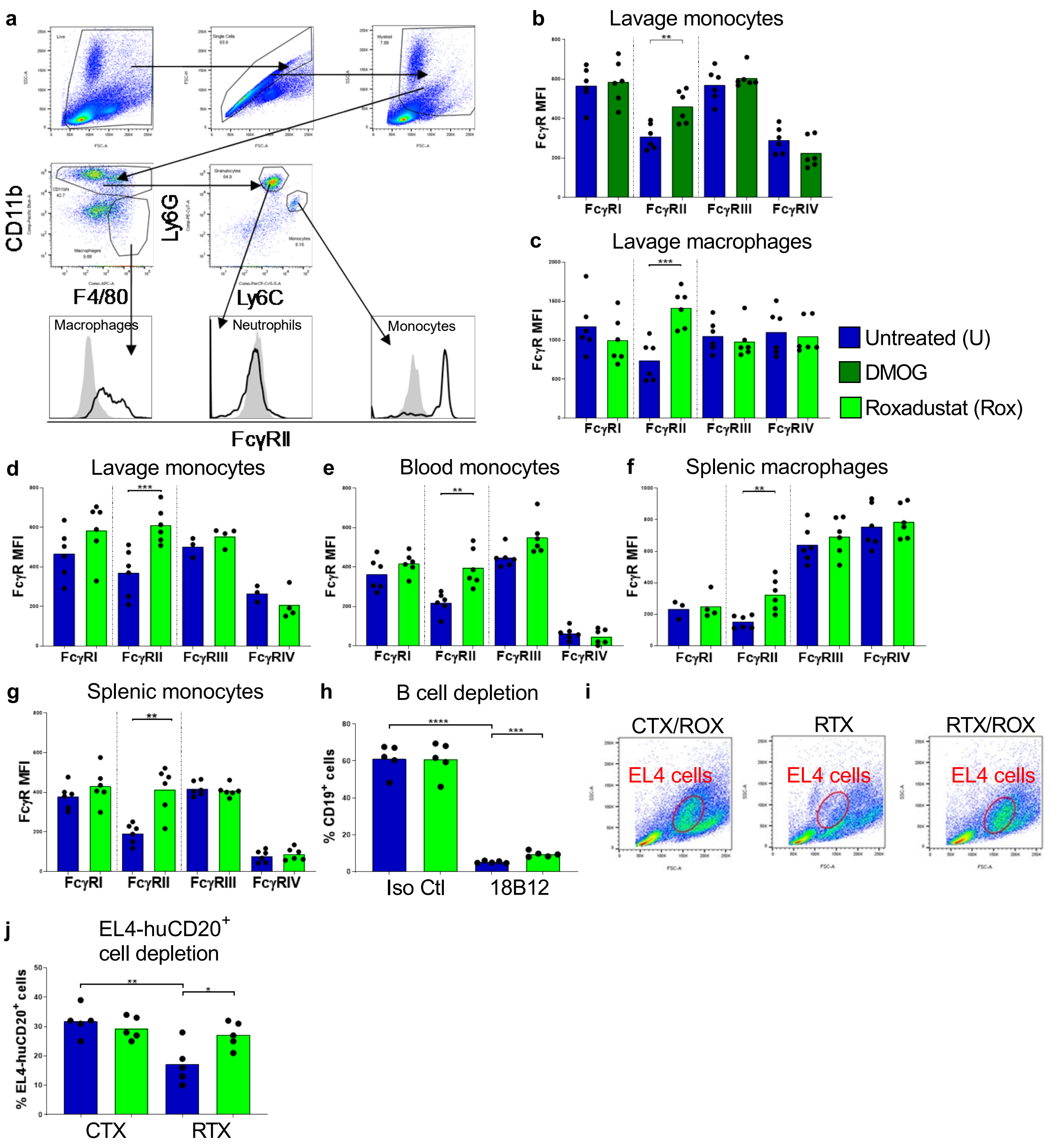

Supplement: Supplementary file 1 — Additional file 1: [file 13046_2022_2294_MOESM1_ESM.pdf]
